# Expansion induced microRNA changes in bone marrow mesenchymal stromal cells reveals interplay between immune regulation and cell cycle

**DOI:** 10.18632/aging.101088

**Published:** 2016-11-09

**Authors:** Lotta Kilpinen, Amarjit Parmar, Dario Greco, Matti Korhonen, Petri Lehenkari, Päivi Saavalainen, Saara Laitinen

**Affiliations:** ^1^ Research and Development, Finnish Red Cross Blood Service, Helsinki, 00310, Finland; ^2^ Research Programs Unit, Immunobiology, University of Helsinki, Helsinki, 00014, Finland; ^3^ Department of Medical and Clinical Genetics, University of Helsinki, Helsinki, 00014, Finland; ^4^ Institute of Biotechnology, University of Helsinki, Helsinki, 00014, Finland; ^5^ Cell Therapy Services, Finnish Red Cross Blood Service, Helsinki, 00310, Finland; ^6^ Department of Anatomy and Cell Biology, Translational and Cancer Research Unit, Medical Research Center, University of Oulu and Oulu University Hospital, Oulu, 90014, Finland

**Keywords:** senescence, microRNA, mesenchymal stromal cells, SOCS3

## Abstract

Mesenchymal stromal cells (MSC) are currently used in many cell based therapies. Prior to use in therapy, extensive expansion is required. We used microarray profiling to investigate expansion induced miRNA and mRNA expression changes of bone marrow MSCs (BM-MSCs) derived from old and young donors. The expression levels of 36 miRNAs were altered in cells derived from the old and respectively 39 miRNAs were altered in cells derived from young donors. Of these, only 12 were differentially expressed in both young and old donor BM-MSCs, and their predicted target mRNAs, were mainly linked to cell proliferation and senescence. Further qPCR verification showed that the expression of miR-1915-3p, miR-1207, miR-3665, and miR-762 correlated with the expansion time at passage 8. Previously described BM-MSC-specific miRNA fingerprints were also detected but these remained unchanged during expansion. Interestingly, members of well-studied miR-17/92 cluster, involved in cell cycle regulation, aging and also development of immune system, were down-regulated specifically in cells from old donors. The role of this cluster in MSC functionality is worth future studies since it links expansion, aging and immune system together.

## INTRODUCTION

MicroRNAs (miRNAs) are small non-coding RNAs (20-22 nucleotides) that regulate gene expression by binding to their target messenger RNA (mRNA), thus resulting in the mRNA's direct translational repression, RNA degradation or a combination of the two. MiRNAs have been shown to have a crucial role in regulating almost all cellular processes including cell growth, proliferation and differentiation [1, 2]. Mesenchymal stem/stromal cells (MSC) have been widely used in various clinical applications, taking advantage of their regenerative and immunomodulative properties [3]. Due to the recognized significance of miRNA regulation in organismal development and stem cell differentiation [4, 5], most studies investigating the role of miRNA regulation in MSCs have focused on the differentiation of MSCs. In addition to the regenerative potential of MSCs, it is actually the immunomodulative properties that make them an attractive candidate for therapeutic applications aiming for treatment of immune system disorders. MSCs are known to interact with many immune system cells, thus modulating immunological responses both *in vitro* and *in vivo* [6]. Several miRNA molecules (including members of miR-17/92 cluster, miR-181 and miR-155) have been shown to be key regulators of various immune responses, such as T-cell development [7].

Mesenchymal stromal cells can be isolated from virtually any tissue [8]. In therapeutic applications, bone marrow (BM), adipose tissue (AT) and cord blood (CB) are the most relevant sources. Although the different origins have been shown to produce MSCs with different immunomodulatory properties [9], the results from various studies are contradictory [10]. Since miRNA regulation is thought to be one of the key players in MSC differentiation and functionality, it has been proposed that MSCs may have a typical miRNA expression pattern which varies only slightly according to the tissue origin [11].

The age of MSC donors and the required *in vitro* expansion of the therapeutic MSCs are considered to be factors that may affect the properties and functionality of MSCs and eventually have a negative effect on the therapeutic outcome [12, 13]. The age-dependent changes in miRNA expression may be one possible explanation for the observed differences between the old and younger donors. Although miRNA expression is relatively stable in the MSCs, some age-dependent changes in miRNA expression have been previously identified [14]. Interestingly, the miRNA expression of hBM-MSCs and hAT-MSCs is differently affected by the donor age [15]. Previous reports also indicate that the replicative senescence is at least partly driven by miRNA regulation [16, 17]. The results are, however, contradictory and the influence of the donor age on the senescence remains unclear.

In this study, we investigated the age-related changes in miRNA regulation in human BM- MSCs by analyzing age-induced changes in miRNA expression profiles together with mRNA expression. We were able to show that, as opposed to mRNA expression levels, miRNA levels underwent only minor changes during continuous passaging. Interestingly, the age of the donor had even less effect on miRNA expression. Therefore, this study shows that miRNA expression is rather robust and BM-MSCs have a distinct miRNA expression pattern that could provide new identifiers for MSCs.

## RESULTS

### MiRNA expression is changed during expansion

MSCs were collected and characterized from five young adult donors (mean age 22.3) and four elderly donors (mean age 76) as previously described [13]. The miRNA expression profiles of BM-MSCs collected from three young adult donors and three old donors were analyzed using Agilent Human miRNA 8×60K microarray (release 16.0), which contains probes for 1,205 human miRNA molecules, 294 of which were expressed in studied samples (Table S1). Let-7a, let 7b, let 7c, let-7e, let-7f, let 7i, miR-100, miR-125b, miR-199 ad miR-21 were the 10 most highly expressed miRNAs in all the samples studied, and all of them are included in the distinct miRNA signature of MSCs [18].

**Figure 1:**
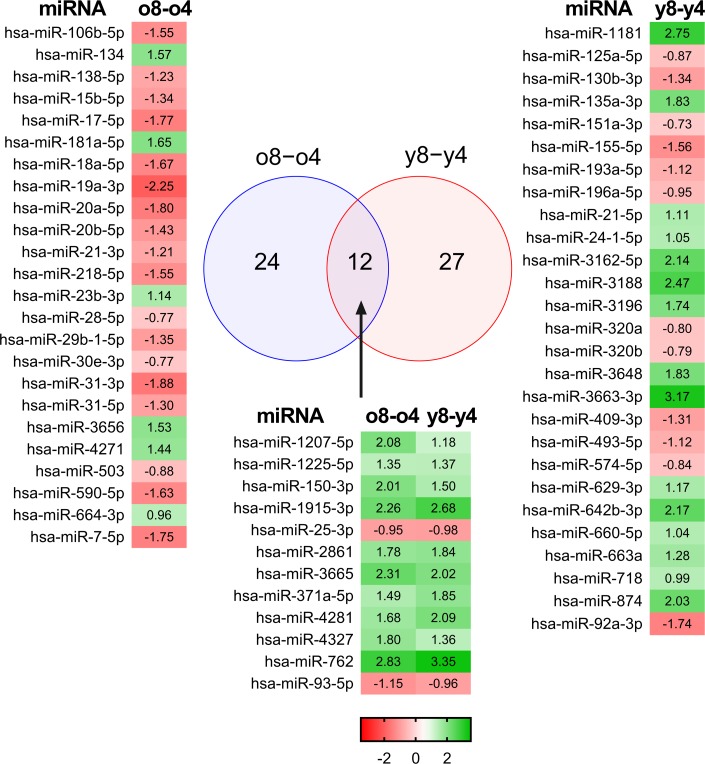
Differentially expressed miRNAs in BM-MSCs Microarray analysis of young and old donors' MSCs found 12 miRNAs whose expression was changed in young and old donors. Fold changes are presented as a heatmap where green color indicates up-regulation and red color indicates down-regulation of the microRNA.

From among the 308 miRNAs, we were able to identify 63 differentially expressed miRNAs (Fig. 1). Interestingly, the miRNA profiles of the young and elderly donors were relatively similar in the early passages. The most prominent changes in miRNA expression were seen towards the late passages. During the expansion of BM-MSCs derived from young donors, the expression of 39 miRNAs was changed, whereas 36 miRNAs were differently expressed in old donors. BM-MSCs from young donors behaved differently during passaging compared to BM-MSCs from the old donors, as both groups shared only 12 differentially regulated miRNAs (Fig. 1). The direction of expression change was also opposite. Our results demonstrate predominant down-regulation of miRNA expression in the old donors' samples and up-regulation in samples from young donors. Of the twelve miRNAs that underwent changes in their expression during expansion in both donor groups, ten were up-regulated and two were down-regulated in both young and old donors.

**Figure 2:**
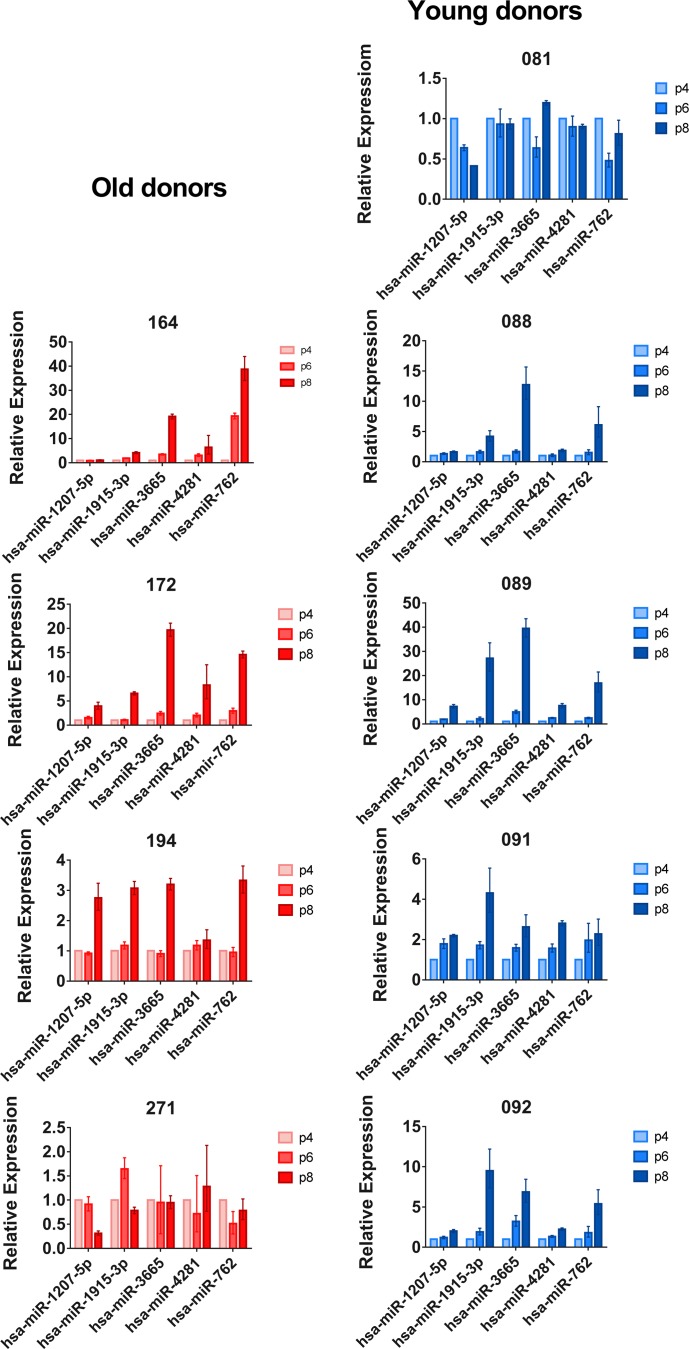
qPCR results of selected miRNAs in five young and four old BM-MSC donors MiRNA expression at passages 4, 6 and 8 was analyzed by qPCR. Results were calculated using the DDC_t_ method and the expression of passage 6 and passage 8 cells was compared to passage 4 for each donor. Results are given as mean + SD of three technical replicates.

In order to validate the microarray results, we performed quantitative PCR for a selected set of differently expressed miRNAs in both young and old donor BM-MSCs (miR-1207, miR-1915-3p, miR-3665, miR4281, and miR-762). Our qPCR results confirmed our microarray results. When individual donors were analyzed separately, we noticed that BM-MSCs from donors 081 (young) and 271 (old) showed no considerable changes during passaging (Fig. 2). These two donors were excluded from further pairwise analyses (Table 1).

**Table 1:** Validation of microarray results with qPCR

miRNA	o8-o4				y8-y4			
	qRT-PCR		microarray		qRT-PCR		microarray	
	FC	p	FC	p	FC	p	FC	p
**miR-762**	3.62	0.04	2.83	0.04	2.83	0.04	3.35	0.05
**miR-3665**	3.41	0.03	2.31	0.04	2.31	0.04	2.02	0.05
**miR-1915-3p**	2.13	0.01	2.26	0.04	2.26	0.04	2.68	0.05
**miR-4281**	2.05	0.06	1.67	0.05	1.67	0.05	2.09	0.06
**miR-1207-5p**	1.17	0.09	2.08	0.03	2.08	0.03	1.18	0.07

MSC donors 081 and 271 were removed from calculation

To investigate whether the up-regulation of miRNA expression is a continuous process we analyzed passages 4, 6, and 8 by qPCR (Fig. 2). We found that miRNA expression increases already at passage 6, but the most dramatic changes were seen from passage 6 to 8. This indicates that the original selection of passage 8 for miRNA microarray analysis, made on the basis of previous findings on proliferation rate, morphology, lipid profile, and gene expression changes, was justified [13].

### MiRNA expression correlates with expansion time

**Figure 3:**
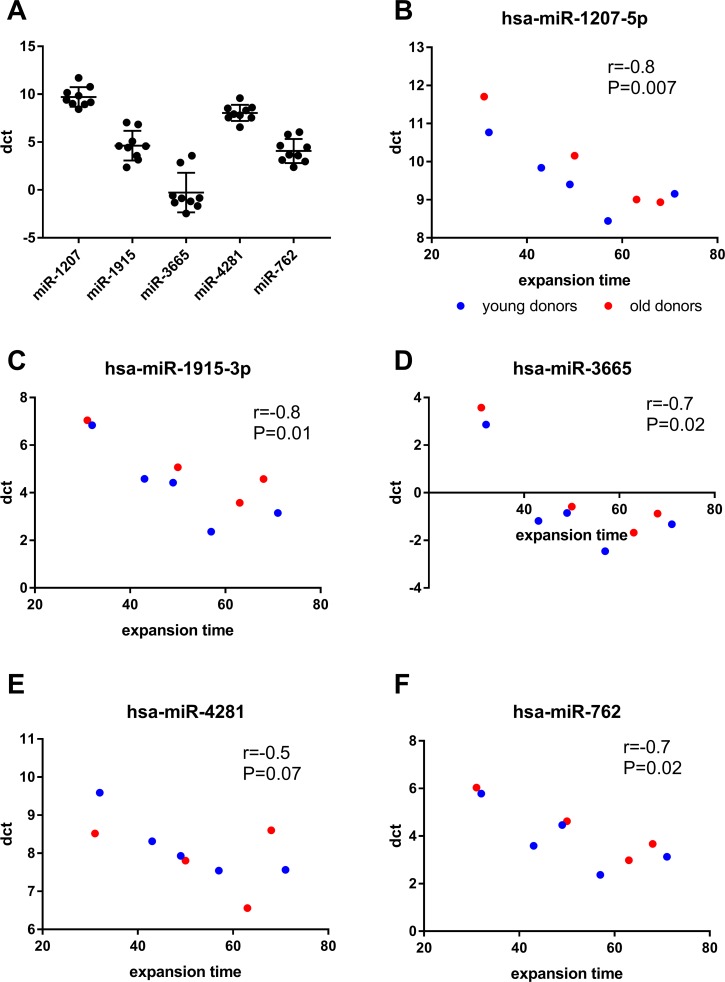
Correlation analyses between miRNA expression and expansion time (**A**) scatter plot of dct values of individual miRNAs at passage 8. (**B-F**) Correlation of dct at passage 8 with the expansion time (days) was calculated using spearman's correlation. Lower dct corresponds to higher miRNA expression.

In our previous work, we have shown that the proliferation capacity and the actual time until passage 8 is reached vary greatly between individual BM-MSC donors [13]. Similar variation was seen in qPCR with delta Ct values between donors at passage 8 (Fig. 3A). To investigate whether the variation in miRNA expression could be explained by expansion time, we performed a correlation analysis. When both young and old donor BM-MSCs were included in the analysis, the dct values of miR-762, miR-1207, miR-1915 and miR-3665 at passage 8 inversely correlated (P<0.05) with the expansion time, whereas for miR-4281, such correlation was only seen in the young donors. (Fig. 3B-F).

### Potential miRNA targets are found mostly in functional pathways of cellular growth, assembly and organization

**Figure 4:**
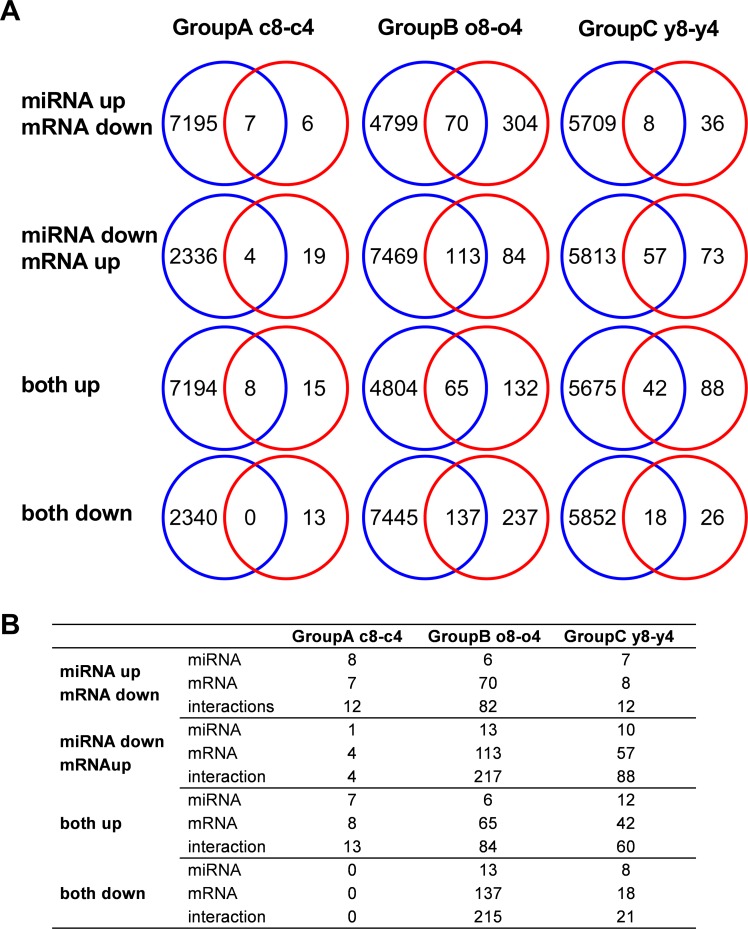
Target prediction analysis of miRNA **(A**) Predicted targets for differentially expressed miRNAs were compared to the differentially expressed mRNAs in the same cells. (**B**) The number of putative miRNA-mRNA interactions and the number of individual miRNAs or mRNAs that account for those interactions.

In order to elucidate the biological processes or pathways that are affected by the changed miRNA expression, we predicted potential target genes through the use of QIAGEN's Ingenuity Pathway Analysis (IPA®, QIAGEN Redwood City, www.qiagen.com/ingenuity). To investigate the overall changes that occur during expansion, we divided our data into three groups. Expression changes that happened in both young and old donor samples were analyzed together (Group A c8-c4), and the unique expression changes in the old donors (Group B o8-o4) and in the young donors (Group C y8-y4) were analyzed separately (Fig. 4).

The number of predicted targets for each individual miRNA ranged from 14 to 2,359 (see Table S2). A functional analysis of predicted target genes of differentially expressed miRNAs showed overrepresentation of cell proliferation and growth, cellular assembly and organization and tissue development (Table 2, Table S2), all of which are related either to the stem cell functions or senescence. In addition, some miRNAs belonging to Group A c8-c4 molecules (such as miR-1225-5p and miR-293-5p) putatively regulate genes that are involved in lipid metabolism. (Table S2).

**Table 2:** Functional classification of miRNA targets

miRNA		Molecular and Cellular function	p-value	number of molecules
*Group A c8-c4*				
miR-3665	**↑**	Cellular Assembly and Organization	9.65E-09-4.21E-02	124/892
miR-17-5p	**↓**	Cell Cycle	1.05E-07-6.68E-03	175/1419
miR-15-5p	**↓**	Cellular Growth and Proliferation	1.97E-08-7.08E-03	369/1419
miR-92a-3p	**↓**	Cellular Assembly and Organization	6.53E-08-2.72E-02	202/1140
miR-92a-3p	**↓**	Tissue Development	6.87E-07-2.67E-02	179/1140
*GroupB o8-o4*				
miR-181a-5p	**↑**	Cellular Growth and Proliferation	1.89E-08-8.62E-03	399/1498
miR-23a-3p	**↑**	Tissue Development	7.15E-10-1.9E-02	157/1506
miR-16-5p	**↓**	Cellular Assembly and Organization	1.75E-08-5.65E-03	271/2020
miR-19a-3p	**↓**	Cellular Assembly and Organization	7.8E-07-1.43E-02	195/1450
miR-19a-3p	**↓**	Cellular Growth and Proliferation	2.77E-07-1.15E-02	382/1450
miR-218-5p	**↓**	Tissue Development	5.15E-07-2.11E-02	143/1279
miR-503-5p	**↓**	Cell Cycle	2.49E-11-7.8E-03	106/772
*Group C y8-y4*				
miR-3180-3p	**↑**	Cellular Assembly and Organization	2.55E-07-2.48E-02	154/1102
miR-3180-3p	**↑**	Tissue Development	3.04E-08-2.48E-02	124/1102
miR-130a-3p	**↓**	Cellular Assembly and Organization	5.44E-07-3.73E-03	179/1253
miR-130a-3p	**↓**	Cellular Growth and Proliferation	9.96E-08-3.68E-03	335/1253
miR-155-5p	**↓**	Tissue Development	8.23E-07-3.91E-03	216/1253
miR-155-5p	**↓**	Cell Cycle	1.97E-07-1.12E-02	94/799

Categories with p-value<E-06 are shown in the table. See Table S2 for the complete list of miRNAs. Arrows indicate up- or down regulation. Number of predicted target genes found in the category/ total number of predicted targets are shown in the number of molecules column.

### Comparison of predicted miRNA targets with differentially expressed mRNA molecules illustrates a complex regulatory network of interactions

To narrow down the list of potential targets, we compared the predicted targets of all up-regulated or all down-regulated miRNAs (Fig. 4A blue circles) with the mRNA expression data derived from the same cells (Fig. 4A red circles). The overlap between these two groups was then investigated in each group (A-C). In group A (c8-c4), the number of down-regulated mRNAs was 13, 7 of which, were predicted to be potential targets of up-regulated miRNAs in the same group. On the other hand, 23 mRNAs were up-regulated, and, we demonstrate in more detail the number of predicted interactions and the number of miRNA and mRNA molecules participating in these interactions in Figure 4B.

**Figure 5:**
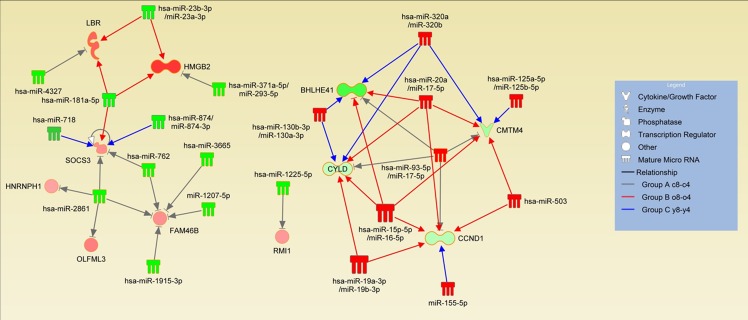
Expansion-induced miRNA-mRNA interactions Interactions of adverse expression changes are shown in the figure. Green color of the miRNA or mRNA represents up-regulation and red color represents down-regulation. Gray arrows indicate interactions in the group A c-8-c4 whereas red color represents the old donors group (Group B o8-o4) and the blue color represents the young donors group (Group C y8-y4). See also Table S3.

Based on the known functions of miRNAs, we focused on the mRNAs that were down-regulated and corresponding miRNAs that were up-regulated and the mRNAs that were up-regulated and corresponding miRNAs that were down-regulated in group A. Seven potential genes were down-regulated (*FAM46B, HMGB2, HNRNPH1,* 4 of them were predicted to be potential targets of down-regulated miRNAs, while 8 were potential targets of up-regulated miRNAs (Fig. 4A). In group B (o8-o4), 374 mRNAs were down-regulated. Of these, 70 were predicted to be potential targets of up-regulated miRNAs, while 137 were potential targets of down-regulated miRNAs in the same group. On the other hand, 197 mRNAs were up-regulated, with 113 predicted to be potential targets of down-regulated miRNAs while 65 were potential targets of down-regulated miRNAs. Lastly, in group C, down-regulation was only seen in 44 mRNAs, 8 of which were predicted as potential targets of up-regulated miRNAs and 18 were predicted targets of down-regulated miRNAs. In contrast, 130 mRNAs were up-regulated. Of these, 57 were predicted to be potential targets of down-regulated miRNAs, while 42 were potential targets of up-regulated miRNAs. Since a single miRNA may target several mRNA molecules and an individual mRNA molecule may be regulated by several different miRNA molecules *LBR, OLFML3, RMI1, SOCS3*) and four potential genes were up-regulated (*BHLHE4, CCND1, CMTM4,* CYLD). In addition to the miRNAs whose expression changed in both groups during expansion, these genes are potential targets of several other miRNAs only found in unique groups B and C, which further supports the finding (Fig. 5, Table S3).

## DISCUSSION

**Figure 6:**
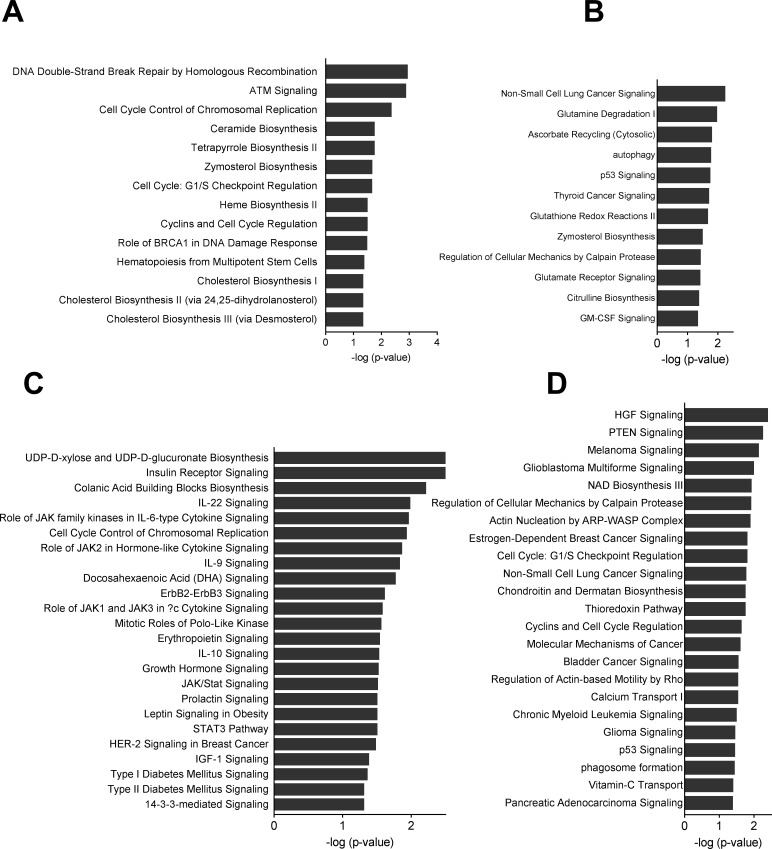
Pathway analyses of miRNA mRNA interactions Canonical pathways were analyzed through the use of QIAGEN's Ingenuity Pathway Analysis. (**A**) Group B o8-o4 down-regulated target mRNAs for up-regulated miRNAs (**B**) Group B o8-o4 up-regulated target mRNAs for down-regulated miRNAs (**C**). Group C y8-y4 down-regulated target mRNAs for up-regulated miRNAs (**D**). Group C y8-y4 up-regulated target mRNAs for up-regulated miRNAs.

We previously reported interesting changes in membrane lipid composition and gene expression during long-term expansion of BM-MSCs [13]. Using the same biological material, we studied more thoroughly changes in both mRNA and miRNA expression and their interactions. The primary aim of the study was to find senescence-related miRNA signatures. For this purpose, our study material was optimal: there was a clear age difference between the young donors (average age 22.3) and the old donors (average age 76). In previous miRNA studies where the importance of the age of the donor has been studied in MSCs, the age difference has not been as pronounced [15, 16]. Despite the clear age difference, miRNA expression was similar in both age groups in the early passage cells (p4). We further investigated whether miRNA and mRNA expression responds differently to expansion in old and young donor BM-MSCs. Previously described miRNA signatures were observed in all the BM-MSCs studied, with expression remaining unchanged during expansion. The expression of many other miRNAs was altered during expansion albeit only moderately. Interestingly, the majority of the differentially expressed miRNAs were unique for either old or young donors, and they regulated different pathways (Fig. 6). Only some of them underwent similar changes in both groups, indicating that expansion influences different processes in BM-MSCs from young and old donors. In our analysis, we mainly concentrated on those miRNAs that were either up- or down-regulated in both groups and compared their predicted targets with our previous mRNA results. This enabled us to find needles in the haystack and narrow down the analyzed miRNAs and their potential mRNA targets.

We successfully validated microarray results with qPCR using selected miRNAs. Interestingly, when all BM-MSCs at passage 8 were analyzed and the expression levels of miRNAs compared with the proliferation data, we found a correlation between dct values and expansion time. Functions of miR-762, miR-1207, miR-1915 miR-3665 and miR-4281 are still largely unknown, but some of them are involved in immunological processes. MiR-762 and miR-1207 are highly expressed in human corneal epithelial cells, where they have been suggested to regulate genes of the innate immune system and contribute to defense against pathogens [19]. When the miRNA expression of different types of T-cells was analyzed, miR-4281 was identified as one potential marker for regulatory T-cells, which play a major role in the suppression of an overheated immune response [20].

The detailed examination of the miRNA results revealed high expression of miRNA members of miR- 17/92 cluster and its two paralogues miR-106b/25 and miR-106a/363. The expression of several miRNAs from the miR-17/92 cluster, including miR-17, miR-18a, miR-19a, and miR-20a, was decreased in the old donors' cells whereas the expression of miR-93, miR-25 and miR- 92a-3p was decreased in the young donors' cells. The miR-17/92 cluster is one of the most extensively studied microRNA clusters. Previous studies have shown down-regulation of miR-17, miR-20a and miR-106a in aging [21]. Direct targeting of p21, one of the key regulators of cell cycle, leads to the attenuation of proliferation and senescence in human fibroblasts [22].We have previously shown that the protein expression of p21 increases during MSC expansion towards senescence. This indicates that at least in the old donors' group, the expansion of BM-MSCs leads to a decrease in the expression of several members of the miR17/92 cluster, which may in turn increase p21 expression, finally resulting in cell cycle arrest.

One of the first identified targets of miR-17/92 cluster is the phosphatase and tensin homolog (PTEN). Although PTEN is not up-regulated in our data, the functional analysis of up-regulated mRNA targets of down-regulated miRNAs reveals enrichment of PTEN signaling and cell cycle related pathways (Fig. 6D). In recent years, several targets for miR17/19 have been experimentally validated. Our results demonstrate that several such genes are up-regulated in BM-MSCs while the corresponding miRNA is down-regulated (Table S4). However, some of the potential targets were also down-regulated in BM-MSCs. This may be a result of autoregulation when miRNA expression is regulated by a transcription factor that is known to be a potential target. For instance, transcription factors E2F1-3 have been shown to directly regulate the expression of miR-20a while miR-20a simultaneously regulates the translation of E2Fs [23, 24].

Even though we demonstrated that expansion effects in the old and young donors' BM-MSCs were different, we were able to identify a set of potentially miRNA regulated genes whose expression changed in both young and old donors' BM-MSCs (Fig. 5, Table S3). *BHLHE41*, *CCND1*, *CMTM4*, and *CYLD* were all up-regulated whereas *FAM46B*, *HMGB2*, *HNRNPH1*, *LBR*, *OLFML3*, *RMI1*, and *SOCS3* were down-regulated. Notably, some of these genes were targets of one common miRNA molecule whereas other mRNAs interact with several miRNA molecules. In addition to the common miRNA molecules, these genes are targets for unique miRNAs whose expression is changed in either young or old donors' BM-MSCs (Fig. 5). BHLHE41 (DEC2) is involved in the control of circadian rhythm, apoptosis, cell differentiation and inflammation [25]. Cyclin D1 (CCND1) controls cell cycle progression while increased senescence is reported to be associated with increased *CCND1*expression levels [26]. Similarly, CMTM4 has also been shown to inhibit cellular growth [27]. CYLD has been proposed to be a master regulator of inflammation and apoptosis [28], further supporting the link between proliferation and immunomodulatory changes.

Biological functions of down-regulated genes, on the other hand, are not well-known. For instance, *FAM46B* was only recently described as active non-canonical poly(A) polymerase and it has been connected to cell cycle regulation based on sequence homology [29]. Since the functions and expression levels of this gene in different cells are not yet known, it remains for future studies to find out the importance of this gene in BM-MSCs.

One of the major biological roles of MSCs stems from their capacity to modulate many immunological processes. Specifically, MSCs suppress T-cell proliferation via indoleamine 2,3-dioxygenase (IDO) [30] and prostaglandin E_2_ (PGE_2_), and they also work together with T-cells in inflammation via purinergic signaling [31]. MiR-21, miR-155 and miRNA family 17/92 are called immunomirs and they regulate T-cell differentiation, function and aging [7]. Of these miRNAs, miR-21 is highly expressed in MSCs and its expression has been shown to be induced by an anti-inflammatory lipid molecule, resolvin D1, which participates in the attenuation of the inflammatory response [32]. MiR-155, on the other hand, inhibits the immunosuppressive capacity of MSCs in mice by reducing iNOS expression [33]. MiR-181 is not included in immunomirs, but it has been shown to be involved in T- and B-cell development [34]. In MSCs, elevated miR-181 expression enhances IL6 and IDO expression, and the capability of MSCs to suppress mitogen activated T-cell proliferation was also attenuated by miR-181 in, at least partly, IL6-dependent manner [35].

In our study, we found several miRNAs (miR-181,- 718,- 874, -762, -2861) with *SOCS3* as a predicted target up-regulated in our study, indicating that this major regulator of inflammation is strongly regulated in MSCs during expansion. Supporting evidence comes from down-regulation of *SOCS3* mRNA expression during expansion and from old donor MSCs where feedback regulator cytokine IL-6 was also up-regulated during expansion. The role of JAK-STAT signaling and SOCS3 is well established not only in regulation of macrophage polarization (M1/M2), but also in Th17 differentiation [36, 37]. The function of SOCS3 in MSCs has not yet been described, but our current observations combined with our previous results showing lowering of immunosuppressive effect during expansion suggest an interesting link between expansion and immunomodulation. Chromatin protein HMGB2 has a crucial role in maintaining stem cell pluripotency [38] and it has been shown to regulate differentiation of MSCs [39]. HMGB1, a HMGB2 isoform, is recognized as an inflammatory cytokine [40] and recently HMGB2′s role in inflammation was also reported at least in neuroinflammation [41].

As a summary, we investigated expansion-induced changes in miRNA expression in BM-MSCs collected from donors of different ages and we could confirm moderate changes in miRNA and mRNA expression that pointed towards interplay between cell cycle, senescence and immunomodulation. As one of the key players in the regulation of the immune system, SOCS3 was targeted by several miRNAs and affected by expansion. Expression of both previously described and functionally unknown miRNAs was changed, a finding which was further supported by the mRNA results. In addition to studies with short term experiments on miRNA/ target gene –changes we still need miRNA profile data to broaden our understanding of the complexity of holistic changes in long lasting processes.

## MATERIALS AND METHODS

### Isolation and cell culture of BM-MSCs

hBM-MSCs were obtained from bone marrow aspirates taken from the iliac crest or upper femur metaphysis of adult patients after written informed consent. All patient protocols were approved by the Ethical Committee of Northern Ostrobothnia Hospital District or Ethical Committee of Hospital District of Helsinki and Uusimaa. The hBM-MSCs were isolated and characterized as previously described [13]. Cells were cultured in minimum essential alpha-medium (αMEM) supplemented with 20mM HEPES, 10% heat inactivated fetal bovine serum (FBS), 2mM L-glutamine and 100 units/mL penicillin and 100 μg/mL streptomycin (all from Gibco, Invitrogen, Paisley, UK). The same serum lot was used throughout the study. The cells were plated at a density of 1000 cells/cm^2^, medium was renewed twice a week and the cells were harvested when 70-80% confluent.

### RNA isolation

RNA was extracted using Qiagen AllPrep DNA/RNA Mini Kit (Cat. no. 80204, Qiagen, CA, USA) and a Qiagen supplementary protocol “Purification of total RNA containing miRNA from animal cells using the RNeasy Plus Mini Kit (cat. no. 74134). In brief, the Qiagen AllPrep DNA/RNA Mini kit was first used to lyse the cells and homogenize the lysate. The DNA was then bound to the AllPrep DNA spin column following the vendor's instructions. The flow-through, after DNA binding, was used to isolate RNA (containing miRNA) using the Qiagen supplementary protocol and the Qiagen RNeasy Plus Mini Kit mentioned above. The quality of RNA was controlled by BioAnalyzer Small RNA method.

### MicroRNA expression profiling

Labeled RNAs (800 ng/sample) were hybridized onto an Agilent Human miRNA 8×60K microarray (release 16.0) and then the slides were washed and scanned according to the manufacturer's recommendations. The raw data files (.txt files) were imported into the R v. 2.13 software [42] and preprocessed by the BioConductor package limma v.3.4.5[43]. After quality control of the data, the median probe intensities were log2 transformed and normalized according to the method of the quantiles [44]. The probes for the same Entrez Genes or lincRNAs (as of 1st January 2012) were averaged.

A linear model including the AGE*PASSAGE + SUBJECT + DYE terms followed by a moderated t-test was utilized for finding the differentially expressed genes in the comparisons of interest (nominal p-value < 0.01).

### Quantitative PCR

For miRNA analyzes, 100 ng total RNA were reverse-transcribed and amplified with real-time PCR using miScript-System including miScript II RT-Kit, miScript SYBR-Green PCR-Kit and miScript Primer Assay (Qiagen) according to the manufacturer's protocol. For endogenous control, RNU6 was used. All PCR reactions were performed in triplicate in 384-well plates and measured by ABI 7900HT detection system Mean values and standard deviations were calculated and fold changes were determined using the ΔΔC_T_ method.

### Target prediction and pathway analysis

Prediction and functional analysis of putative targets for selected miRNAs was performed through the use of QIAGEN's Ingenuity Pathway Analysis (IPA QIAGEN Redwood City, www.qiagen.com/ingenuity)

### Statistical analysis

Results are represented as mean±standard deviation. Pairwise t-test (one tailed) was used to compare the groups, and p-value <0.05 was considered statistically significant. Non-parametric Spearman correlation was used to calculate correlation between miRNA expression and proliferation of MSCs. All analyses were performed with GraphPad Prism 7.00 software (GraphPad Software Inc. La Jolla, CA, USA).

## SUPPLEMENTARY MATERIAL TABLES



## References

[1] Bartel DP (2009). MicroRNAs: target recognition and regulatory functions. Cell.

[2] Ameres SL, Zamore PD (2013). Diversifying microRNA sequence and function. Nat Rev Mol Cell Biol.

[3] Sharma RR, Pollock K, Hubel A, McKenna D (2014). Mesenchymal stem or stromal cells: a review of clinical applications and manufacturing practices. Transfusion.

[4] Ivey KN, Muth A, Arnold J, King FW, Yeh RF, Fish JE, Hsiao EC, Schwartz RJ, Conklin BR, Bernstein HS, Srivastava D (2008). MicroRNA regulation of cell lineages in mouse and human embryonic stem cells. Cell Stem Cell.

[5] Ong SG, Lee WH, Kodo K, Wu JC (2015). MicroRNA-mediated regulation of differentiation and trans-differentiation in stem cells. Adv Drug Deliv Rev.

[6] Uccelli A, de Rosbo NK (2015). The immunomodulatory function of mesenchymal stem cells: mode of action and pathways. Ann N Y Acad Sci.

[7] Kroesen BJ, Teteloshvili N, Smigielska-Czepiel K, Brouwer E, Boots AM, van den Berg A, Kluiver J (2015). Immuno-miRs: critical regulators of T-cell development, function and ageing. Immunology.

[8] da Silva Meirelles L, Chagastelles PC, Nardi NB, Meirelles LdS (2006). Mesenchymal stem cells reside in virtually all post-natal organs and tissues. J Cell Sci.

[9] Di Trapani M, Bassi G, Ricciardi M, Fontana E, Bifari F, Pacelli L, Giacomello L, Pozzobon M, Féron F, De Coppi P, Anversa P, Fumagalli G, Decimo I (2013). Comparative study of immune regulatory properties of stem cells derived from different tissues. Stem Cells Dev.

[10] Mattar P, Bieback K (2015). Comparing the immune-modulatory properties of bone marrow, adipose tissue, and birth-associated tissue mesen-chymal stromal cells. Front Immunol.

[11] Ragni E, Montemurro T, Montelatici E, Lavazza C, Viganò M, Rebulla P, Giordano R, Lazzari L (2013). Differential microRNA signature of human mesenchymal stem cells from different sources reveals an “environmental-niche memory” for bone marrow stem cells. Exp Cell Res.

[12] von Bahr L, Sundberg B, Lönnies L, Sander B, Karbach H, Hägglund H, Ljungman P, Gustafsson B, Karlsson H, Le Blanc K, Ringdén O (2012). Long-term complications, immunologic effects, and role of passage for outcome in mesenchymal stromal cell therapy. Biol Blood Marrow Transplant.

[13] Kilpinen L, Tigistu-Sahle F, Oja S, Greco D, Parmar A, Saavalainen P, Nikkilä J, Korhonen M, Lehenkari P, Käkelä R, Laitinen S (2013). Aging bone marrow mesenchymal stromal cells have altered membrane glycerophospholipid composition and functionality. J Lipid Res.

[14] Candini O, Spano C, Murgia A, Grisendi G, Veronesi E, Piccinno MS, Ferracin M, Negrini M, Giacobbi F, Bambi F, Horwitz EM, Conte P, Paolucci P, Dominici M (2015). Mesenchymal progenitors aging highlights a miR-196 switch targeting HOXB7 as master regulator of proliferation and osteogenesis. Stem Cells.

[15] Pandey AC, Semon JA, Kaushal D, O'Sullivan RP, Glowacki J, Gimble JM, Bunnell BA (2011). MicroRNA profiling reveals age-dependent differential expression of nuclear factor κB and mitogen-activated protein kinase in adipose and bone marrow-derived human mesenchymal stem cells. Stem Cell Res Ther.

[16] Wagner W, Horn P, Castoldi M, Diehlmann A, Bork S, Saffrich R, Benes V, Blake J, Pfister S, Eckstein V, Ho AD (2008). Replicative senescence of mesenchymal stem cells: a continuous and organized process. PLoS One.

[17] Yoo JK, Kim CH, Jung HY, Lee DR, Kim JK (2014). Discovery and characterization of miRNA during cellular senescence in bone marrow-derived human mesenchymal stem cells. Exp Gerontol.

[18] Clark EA, Kalomoiris S, Nolta JA, Fierro FA (2014). Concise review: MicroRNA function in multipotent mesenchymal stromal cells. Stem Cells.

[19] Mun J, Tam C, Chan G, Kim JH, Evans D, Fleiszig S (2013). MicroRNA-762 is upregulated in human corneal epithelial cells in response to tear fluid and Pseudomonas aeruginosa antigens and negatively regulates the expression of host defense genes encoding RNase7 and ST2. PLoS One.

[20] Smigielska-Czepiel K, van den Berg A, Jellema P, van der Lei RJ, Bijzet J, Kluiver J, Boots AM, Brouwer E, Kroesen BJ (2014). Comprehensive analysis of miRNA expression in T-cell subsets of rheumatoid arthritis patients reveals defined signatures of naive and memory Tregs. Genes Immun.

[21] Hackl M, Brunner S, Fortschegger K, Schreiner C, Micutkova L, Mück C, Laschober GT, Lepperdinger G, Sampson N, Berger P, Herndler-Brandstetter D, Wieser M, Kühnel H (2010). miR-17, miR-19b, miR-20a, and miR-106a are down-regulated in human aging. Aging Cell.

[22] Gómez-Cabello D, Adrados I, Gamarra D, Kobayashi H, Takatsu Y, Takatsu K, Gil J, Palmero I (2013). DGCR8-mediated disruption of miRNA biogenesis induces cellular senescence in primary fibroblasts. Aging Cell.

[23] Sylvestre Y, De Guire V, Querido E, Mukhopadhyay UK, Bourdeau V, Major F, Ferbeyre G, Chartrand P (2007). An E2F/miR-20a autoregulatory feedback loop. J Biol Chem.

[24] Hemming S, Cakouros D, Vandyke K, Davis MJ, Zannettino AC, Gronthos S (2016). Identification of novel EZH2 targets regulating osteogenic differentiation in mesenchymal stem cells. Stem Cells Dev.

[25] Bigot P, Colli LM, Machiela MJ, Jessop L, Myers TA, Carrouget J, Wagner S, Roberson D, Eymerit C, Henrion D, Chanock SJ (2016). Functional characterization of the 12p12.1 renal cancer-susceptibility locus implicates BHLHE41. Nat Commun.

[26] Berenstein R, Blau O, Nogai A, Waechter M, Slonova E, Schmidt-Hieber M, Kunitz A, Pezzutto A, Doerken B, Blau IW (2015). Multiple myeloma cells alter the senescence phenotype of bone marrow mesenchymal stromal cells under participation of the DLK1-DIO3 genomic region. BMC Cancer.

[27] Plate M, Li T, Wang Y, Mo X, Zhang Y, Ma D, Han W (2010). Identification and characterization of CMTM4, a novel gene with inhibitory effects on HeLa cell growth through Inducing G2/M phase accumulation. Mol Cells.

[28] Mathis BJ, Lai Y, Qu C, Janicki JS, Cui T (2015). CYLD-mediated signaling and diseases. Curr Drug Targets.

[29] Kuchta K, Muszewska A, Knizewski L, Steczkiewicz K, Wyrwicz LS, Pawlowski K, Rychlewski L, Ginalski K (2016). FAM46 proteins are novel eukaryotic non-canonical poly(A) polymerases. Nucleic Acids Res.

[30] Meisel R, Zibert A, Laryea M, Göbel U, Däubener W, Dilloo D (2004). Human bone marrow stromal cells inhibit allogeneic T-cell responses by indoleamine 2,3-dioxygenase-mediated tryptophan degradation. Blood.

[31] Kerkelä E, Laitinen A, Räbinä J, Valkonen S, Takatalo M, Larjo A, Veijola J, Lampinen M, Siljander P, Lehenkari P, Alfthan K, Laitinen S (2016). Adenosinergic immunosuppression by human mesenchymal stromal cells (MSCs) requires co-operation with T cells. Stem Cells.

[32] Recchiuti A, Krishnamoorthy S, Fredman G, Chiang N, Serhan CN (2011). MicroRNAs in resolution of acute inflammation: identification of novel resolvin D1-miRNA circuits. FASEB J.

[33] Xu C, Ren G, Cao G, Chen Q, Shou P, Zheng C, Du L, Han X, Jiang M, Yang Q, Lin L, Wang G, Yu P (2013). miR-155 regulates immune modulatory properties of mesenchymal stem cells by targeting TAK1-binding protein 2. J Biol Chem.

[34] Ebert PJ, Jiang S, Xie J, Li QJ, Davis MM (2009). An endogenous positively selecting peptide enhances mature T cell responses and becomes an autoantigen in the absence of microRNA miR-181a. Nat Immunol.

[35] Liu L, Wang Y, Fan H, Zhao X, Liu D, Hu Y, Kidd AR, Bao J, Hou Y (2012). MicroRNA-181a regulates local immune balance by inhibiting proliferation and immunosuppressive properties of mesenchymal stem cells. Stem Cells.

[36] Chen Z, Laurence A, Kanno Y, Pacher-Zavisin M, Zhu BM, Tato C, Yoshimura A, Hennighausen L, O'Shea JJ (2006). Selective regulatory function of Socs3 in the formation of IL-17-secreting T cells. Proc Natl Acad Sci USA.

[37] Qin H, Holdbrooks AT, Liu Y, Reynolds SL, Yanagisawa LL, Benveniste EN (2012). SOCS3 deficiency promotes M1 macrophage polarization and inflammation. J Immunol.

[38] Campbell PA, Rudnicki MA (2013). Oct4 interaction with Hmgb2 regulates Akt signaling and pluripotency. Stem Cells.

[39] Taniguchi N, Caramés B, Hsu E, Cherqui S, Kawakami Y, Lotz M (2011). Expression patterns and function of chromatin protein HMGB2 during mesenchymal stem cell differentiation. J Biol Chem.

[40] Lotze MT, Tracey KJ (2005). High-mobility group box 1 protein (HMGB1): nuclear weapon in the immune arsenal. Nat Rev Immunol.

[41] Lee S, Nam Y, Koo JY, Lim D, Park J, Ock J, Kim J, Suk K, Park SB (2014). A small molecule binding HMGB1 and HMGB2 inhibits microglia-mediated neuroinflam-mation. Nat Chem Biol.

[42] R Core Team (2015). R: A language and environment for statistical computing.

[43] Smyth GK, Gentleman R, Carey V, Duboit S, Irizarry R, Huber W (2005). Limma: Linear models for microarray data. Bioinformatics and computational biology solutions using R and bioconductor.

[44] Bolstad BM, Irizarry RA, Astrand M, Speed TP (2003). A comparison of normalization methods for high density oligonucleotide array data based on variance and bias. Bioinformatics.

