# Supplementary material for: Expansion induced microRNA changes in bone marrow mesenchymal stromal cells reveals interplay between immune regulation and cell cycle
Source: Aging (Albany NY). 2016 Nov 9;8(11):2799–812. doi: 10.18632/aging.101088 (PMC5191871; doi:10.18632/aging.101088)
Supplement: Supplementary file 1 [file aging-08-2799-s001.pdf]

SUPPLEMENTARY MATERIAL

Supplemental Table S1.

Supplemental Table S2.

Supplemental Table S3.

Supplemental Table S4.

References.

SUPPLEMENTAL TABLE S1

	young donors								
	088_4	089_4	092_4	average	088_8	089_8	092_8	average	FC y8-y4
hsa-let-7a-3p	2.719873	2.794604	2.734603	2.749694	2.582188	2.622401	2.5322	2.57893	-0.17076
hsa-let-7a-5p	13.35864	13.49498	12.98383	13.27915	13.23577	13.42483	13.38644	13.34901	0.069862
hsa-let-7b-5p	12.44941	12.66935	11.86678	12.32851	12.16468	11.98154	12.10075	12.08232	-0.24619
hsa-let-7c	10.68915	11.01742	10.22097	10.64251	10.11127	9.914012	9.978104	10.00113	-0.64139
hsa-let-7d-5p	9.610066	9.651263	9.047516	9.436282	8.961107	8.905614	8.785871	8.884197	-0.55208
hsa-let-7e-5p	10.90923	11.48309	10.2392	10.87717	10.18749	10.19599	10.24125	10.20825	-0.66893
hsa-let-7f-5p	12.38744	12.49563	12.10414	12.32907	12.33916	12.38648	12.30713	12.34426	0.015186
hsa-let-7g-5p	10.08846	9.75215	9.848763	9.896457	9.788175	9.581752	9.589349	9.653092	-0.24336
hsa-let-7i-5p	12.14966	12.00919	11.58952	11.91612	11.84798	12.41336	12.15545	12.13893	0.222804
hsa-miR-100-5p	10.86835	10.62857	11.38819	10.96171	11.21258	10.33574	11.53199	11.02677	0.065064
hsa-miR-101-3p	3.83795	3.055521	5.105951	3.999807	5.502548	5.690154	5.778991	5.657231	1.657424
hsa-miR-103a-3p	9.450783	8.993987	9.021307	9.155359	9.271976	8.974103	8.914099	9.053393	-0.10197
hsa-miR-106b-5p	6.610698	5.513167	7.352613	6.492159	6.81402	6.264309	6.401832	6.493387	0.001228
hsa-miR-107	9.209377	8.77902	8.797662	8.928686	9.017126	8.769342	8.723219	8.836563	-0.09212
hsa-miR-10a-5p	7.484381	7.432793	6.801754	7.239643	6.698161	6.628262	6.60428	6.643568	-0.59607
hsa-miR-10b-5p	8.013657	7.619377	7.82982	7.820951	8.077329	7.381368	7.808486	7.755728	-0.06522
hsa-miR-1181	4.843	4.936079	5.324407	5.034495	6.817115	8.82298	7.703712	7.781269	2.746773
hsa-miR-1185-5p	3.428738	3.038093	3.576304	3.347712	3.308842	3.157959	3.629475	3.365425	0.017714
hsa-miR-1202	6.482164	6.792547	6.428911	6.567874	7.036783	8.200518	7.076328	7.437876	0.870002
hsa-miR-1207-5p	7.441455	8.166368	7.062725	7.556849	8.528224	9.066957	8.604778	8.73332	1.17647
hsa-miR-1224-5p	3.316422	3.805809	3.402086	3.508106	4.734677	6.751582	4.719517	5.401925	1.89382
hsa-miR-1225-3p	3.811035	3.805511	3.506099	3.707548	3.758772	4.164468	4.029093	3.984111	0.276562
hsa-miR-1225-5p	6.524754	7.026751	6.765203	6.772236	8.01558	8.366155	8.039599	8.140445	1.368209
hsa-miR-1226-5p	3.073904	3.26355	3.129731	3.155729	3.720683	4.068035	3.829806	3.872841	0.717113
hsa-miR-1228-3p	4.400351	4.757446	4.012475	4.39009	3.885241	3.910587	4.0431	3.946309	-0.44378
hsa-miR-1234	4.709192	5.066425	4.459963	4.745193	5.132461	5.630401	5.565671	5.442844	0.697651
hsa-miR-1238	3.899974	4.081734	3.596658	3.859455	3.337047	3.169428	3.532399	3.346292	-0.51316
hsa-miR-1246	3.876834	4.572971	4.892776	4.447527	4.941403	4.047918	4.887583	4.625635	0.178108
hsa-miR-1249	3.062211	2.919283	2.993437	2.991644	3.46116	3.519028	3.399624	3.459937	0.468293
hsa-miR-125a-3p	3.847501	4.050795	4.011333	3.969877	4.430015	5.047241	4.479029	4.652095	0.682218
hsa-miR-125a-5p	8.72031	8.748311	8.132964	8.533862	7.691574	7.529307	7.767463	7.662781	-0.87108
hsa-miR-125b-5p	12.91385	12.48518	12.49236	12.63047	12.73421	12.53148	12.51296	12.59288	-0.03758
hsa-miR-1260a	10.29527	10.15716	10.3566	10.26968	9.890952	9.074798	9.474214	9.479988	-0.78969
hsa-miR-1260b	9.970619	9.871723	9.923272	9.921871	9.522614	8.544926	9.009338	9.025626	-0.89625

hsa-miR-1268a	3.776327	4.602463	4.092728	4.157173	4.934361	5.521765	4.90167	5.119265	0.962093
hsa-miR-1271-5p	3.455534	3.575147	3.794926	3.608536	3.352619	3.157336	3.337866	3.282607	-0.32593
hsa-miR-127-3p	8.019608	8.302883	7.452763	7.925085	6.578137	6.458297	7.530985	6.855807	-1.06928
hsa-miR-1274a_v16	9.237266	8.52061	9.423404	9.060427	8.799696	8.118638	8.543004	8.487113	-0.57331
hsa-miR-1274b_v16	12.4356	11.99363	12.9315	12.45358	12.52472	11.47562	12.11602	12.03879	-0.41479
hsa-miR-1275	4.064902	4.677715	4.31307	4.351896	4.629799	5.415945	4.562889	4.869545	0.517649
hsa-miR-128	5.112469	5.231866	4.695842	5.013392	4.558855	4.352584	4.331585	4.414341	-0.59905
hsa-miR-1280	6.571224	6.598438	6.382364	6.517342	6.006439	6.077732	6.020175	6.034782	-0.48256
hsa-miR-1288	4.659126	5.133943	4.016697	4.603255	3.872693	3.804668	3.956074	3.877811	-0.72544
hsa-miR-1290	3.388808	3.706294	3.891686	3.662262	4.094877	4.362865	4.250149	4.235963	0.573701
hsa-miR-1305	6.266999	6.990564	5.823669	6.36041	5.248696	5.335003	5.055711	5.213137	-1.14727
hsa-miR-130a-3p	9.530411	8.803794	9.705387	9.346531	9.257644	8.513489	9.036218	8.935784	-0.41075
hsa-miR-130b-3p	6.956965	6.612775	6.821447	6.797062	6.229113	4.938088	5.192084	5.453095	-1.34397
hsa-miR-132-3p	5.924955	5.963763	4.510565	5.466428	5.4336	4.243066	4.238607	4.638424	-0.828
hsa-miR-132-5p	3.713741	3.546955	3.327373	3.529356	3.67945	3.157959	3.167875	3.335095	-0.19426
hsa-miR-134	5.23906	5.885754	4.778518	5.301111	5.824157	6.385844	6.095238	6.101746	0.800636
hsa-miR-135a-3p	3.054244	2.94827	3.467762	3.156759	4.124506	6.00862	4.823639	4.985588	1.828829
hsa-miR-136-3p	3.347362	2.986593	4.642678	3.658878	4.013729	4.010046	5.027315	4.350363	0.691486
hsa-miR-136-5p	3.710731	3.609151	6.387141	4.569008	5.516544	5.43431	7.037906	5.996253	1.427245
hsa-miR-137	7.208539	6.492372	7.521816	7.074242	7.945436	8.151913	8.157718	8.085022	1.01078
hsa-miR-138-5p	4.5279	3.958259	4.041067	4.175742	3.35722	3.364402	3.471874	3.397832	-0.77791
hsa-miR-140-3p	8.303886	7.607701	7.7706	7.894062	7.315451	7.660777	7.888049	7.621426	-0.27264
hsa-miR-140-5p	8.372978	7.520247	8.951189	8.281471	8.767173	9.09466	9.178533	9.013455	0.731984
hsa-miR-143-3p	5.920239	5.501289	6.727235	6.049588	6.873328	6.855055	7.109969	6.946117	0.896529
hsa-miR-143-5p	4.481123	4.549555	4.120997	4.383892	4.287981	4.246659	4.254085	4.262908	-0.12098
hsa-miR-145-3p	5.224934	4.813089	4.845287	4.961103	4.943806	4.872278	5.008391	4.941492	-0.01961
hsa-miR-145-5p	10.07423	10.04316	9.324453	9.813947	9.700843	9.885324	9.963056	9.849741	0.035794
hsa-miR-146b-5p	3.526956	3.374964	4.387215	3.763045	3.958452	4.016826	4.497151	4.157476	0.394431
hsa-miR-148a-3p	6.157357	5.267068	6.140256	5.854893	6.452892	5.376226	4.304678	5.377932	-0.47696
hsa-miR-148b-3p	4.535621	4.170744	4.861501	4.522622	5.193938	5.249361	4.956032	5.13311	0.610488
hsa-miR-149-5p	4.269722	4.089078	3.751143	4.036647	3.867315	3.463527	3.536325	3.622389	-0.41426
hsa-miR-150-3p	5.428351	5.883584	5.484482	5.598806	6.833358	7.436199	7.016869	7.095475	1.49667
hsa-miR-151a-3p	5.71595	5.664837	5.403984	5.594924	5.127747	4.766847	4.708985	4.86786	-0.72706
hsa-miR-151a-5p	7.781686	7.877672	7.346405	7.668588	7.57307	7.448142	7.354604	7.458605	-0.20998
hsa-miR-152	5.514659	5.260456	5.528471	5.434529	5.783721	6.028291	5.938864	5.916959	0.48243
hsa-miR-154-3p	4.300861	3.592944	5.496742	4.463516	4.118414	3.665265	4.912775	4.232152	-0.23136
hsa-miR-154-5p	4.071686	3.700642	4.260883	4.01107	3.790792	3.6482	4.511774	3.983589	-0.02748

hsa-miR-155-5p	5.058467	5.28227	5.099103	5.146613	4.090119	3.286759	3.374513	3.583797	-1.56282
hsa-miR-15a-5p	7.895664	7.273721	8.781828	7.983738	8.426686	8.61095	8.695111	8.577582	0.593845
hsa-miR-15b-5p	9.74023	8.887874	9.723892	9.450665	8.974243	8.217796	8.692777	8.628272	-0.82239
hsa-miR-16-2-3p	2.734196	2.807499	2.97761	2.839768	2.807691	2.649109	2.662981	2.706594	-0.13317
hsa-miR-16-5p	10.46673	9.549448	10.56735	10.19451	10.10902	9.809992	10.20389	10.04097	-0.15354
hsa-miR-17-5p	6.878811	6.033462	7.005878	6.639383	6.392042	4.856838	5.036883	5.428588	-1.2108
hsa-miR-181a-2-3p	3.543954	3.734835	3.573494	3.617428	3.506994	3.602692	3.770969	3.626885	0.009457
hsa-miR-181a-3p	3.316422	3.365347	4.002964	3.561578	4.076968	3.750297	3.874437	3.900568	0.33899
hsa-miR-181a-5p	7.931165	8.790129	7.020442	7.913912	8.244558	8.580895	8.149497	8.324983	0.411071
hsa-miR-181b-5p	6.208639	6.980749	5.608207	6.265865	5.764016	5.650146	5.488332	5.634164	-0.6317
hsa-miR-181c-5p	3.346559	3.673475	3.211655	3.410563	3.859537	3.986511	3.671576	3.839208	0.428645
hsa-miR-181d	3.479317	3.809747	3.23152	3.506861	3.41605	3.345331	3.273775	3.345052	-0.16181
hsa-miR-185-5p	5.372456	4.709239	5.409188	5.163627	5.325269	5.28313	5.044989	5.217796	0.054169
hsa-miR-186-5p	4.074162	3.807349	3.856804	3.912772	4.355363	4.36336	4.096713	4.271812	0.35904
hsa-miR-187-5p	2.714637	2.725502	2.942108	2.794082	3.492503	3.92847	3.568182	3.663052	0.86897
hsa-miR-188-5p	3.674741	4.170261	4.026787	3.957263	4.581257	4.648718	4.218843	4.482939	0.525676
hsa-miR-18a-5p	3.42964	2.911603	4.384143	3.575129	3.871151	3.124644	3.191406	3.395734	-0.17939
hsa-miR-18b-5p	2.901843	2.681401	3.109045	2.89743	2.867672	2.622567	2.833067	2.774435	-0.12299
hsa-miR-191-3p	3.966047	4.261881	3.627603	3.951844	3.477059	3.151136	3.577955	3.40205	-0.54979
hsa-miR-1914-3p	5.141638	5.63864	4.536529	5.105602	4.196914	4.316963	4.192068	4.235315	-0.87029
hsa-miR-1915-3p	7.062463	7.465281	7.545487	7.357743	9.682841	10.58971	9.851048	10.0412	2.683457
hsa-miR-193a-3p	5.185885	4.576966	7.172056	5.644969	6.81643	6.833808	7.711366	7.120535	1.475566
hsa-miR-193a-5p	6.664395	7.176361	6.073171	6.637975	5.559486	5.380335	5.62642	5.52208	-1.1159
hsa-miR-193b-3p	7.870009	7.480086	7.603182	7.651092	6.831698	7.003168	7.377061	7.070642	-0.58045
hsa-miR-193b-5p	3.507855	3.604861	3.419148	3.510621	3.260978	3.339469	3.412954	3.337801	-0.17282
hsa-miR-195-5p	6.85214	6.06023	6.794344	6.568905	6.14029	6.284041	6.708616	6.377649	-0.19126
hsa-miR-196a-5p	7.814285	8.125118	7.191021	7.710141	6.554202	6.913177	6.822185	6.763188	-0.94695
hsa-miR-196b-5p	7.328438	7.498156	6.931804	7.252799	6.733118	6.909739	6.498831	6.713896	-0.5389
hsa-miR-1973	4.27591	5.374035	4.265755	4.638567	4.274189	4.521981	4.152826	4.316332	-0.32223
hsa-miR-197-3p	4.385696	4.935362	3.933315	4.418125	3.773989	4.047127	3.922317	3.914478	-0.50365
hsa-miR-199a-3p	11.41652	11.22191	11.79231	11.47692	11.89767	12.05387	11.94008	11.96387	0.486958
hsa-miR-199a-5p	8.786655	7.762954	9.75717	8.768926	9.862832	9.966349	10.0455	9.958227	1.189301
hsa-miR-199b-5p	3.856014	3.581637	6.067121	4.501591	5.588255	4.138284	4.554398	4.760312	0.258721
hsa-miR-19a-3p	5.958708	4.595275	7.654252	6.069412	6.704707	5.223489	6.087746	6.005314	-0.0641
hsa-miR-19b-3p	7.871722	6.893216	9.160276	7.975071	8.453129	6.972119	7.836917	7.754055	-0.22102
hsa-miR-20a-5p	7.697629	6.760747	7.935759	7.464712	7.139454	5.816953	6.149652	6.368686	-1.09603
hsa-miR-20b-5p	5.074741	4.26977	5.271564	4.872025	4.780407	3.92171	3.910675	4.204264	-0.66776

hsa-miR-210	6.719714	6.217712	3.839914	5.592447	5.457928	5.262758	4.768231	5.162972	-0.42947
hsa-miR-21-3p	6.358858	5.533113	6.92585	6.272607	6.322387	5.494044	6.103894	5.973441	-0.29917
hsa-miR-214-3p	9.47082	9.679566	9.460138	9.536841	9.043606	9.124209	9.024597	9.064137	-0.4727
hsa-miR-214-5p	3.922116	3.427538	4.654114	4.001256	4.135313	4.053353	4.035889	4.074852	0.073596
hsa-miR-21-5p	13.96997	13.75283	14.56731	14.0967	15.03276	15.29422	15.3059	15.21096	1.114256
hsa-miR-218-5p	3.273548	2.929926	3.98804	3.397171	3.470448	2.996262	3.108064	3.191592	-0.20558
hsa-miR-221-3p	10.98638	10.65009	11.02638	10.88762	10.41638	10.34184	10.4689	10.40904	-0.47858
hsa-miR-221-5p	6.893287	6.59095	6.779095	6.754444	6.304454	6.071629	6.510862	6.295648	-0.4588
hsa-miR-222-3p	6.76313	6.034917	6.769315	6.522454	5.885395	5.494494	6.136829	5.838906	-0.68355
hsa-miR-22-3p	11.65665	11.70291	11.24975	11.53644	11.73026	11.84679	11.72464	11.76723	0.23079
hsa-miR-224-5p	7.356556	7.678116	5.982449	7.005707	5.878509	3.804681	4.956928	4.88004	-2.12567
hsa-miR-22-5p	5.697819	6.015743	5.905051	5.872871	5.664336	5.857327	5.776292	5.765985	-0.10689
hsa-miR-23a-3p	12.17958	12.2491	11.99605	12.14158	12.4664	12.75112	12.58813	12.60188	0.460305
hsa-miR-23b-3p	10.95271	11.00404	10.51976	10.8255	11.04904	11.51215	11.11971	11.22697	0.401466
hsa-miR-23b-5p	2.882905	3.033006	3.096536	3.004149	3.161001	3.10587	3.209404	3.158758	0.154609
hsa-miR-24-1-5p	3.082495	2.920324	3.787315	3.263378	4.09258	4.405579	4.445579	4.314579	1.051201
hsa-miR-24-3p	11.44104	11.30868	11.3875	11.37907	11.82958	12.02401	11.90552	11.9197	0.540632
hsa-miR-25-3p	7.380178	7.045692	7.208375	7.211415	6.47619	6.00702	6.215834	6.233014	-0.9784
hsa-miR-26a-5p	9.335816	8.557497	9.105339	8.999551	9.126207	8.892466	9.1377	9.052124	0.052574
hsa-miR-26b-5p	8.567582	8.202588	7.993678	8.254616	8.501326	8.273479	8.318422	8.364409	0.109793
hsa-miR-27a-3p	10.23893	9.374991	11.40057	10.33816	11.5764	11.55524	11.6926	11.60808	1.269917
hsa-miR-27b-3p	10.45311	9.732239	10.64966	10.27834	10.74007	10.82668	10.96854	10.8451	0.566761
hsa-miR-28-5p	5.651749	5.088921	5.679233	5.473301	5.594486	5.387978	5.490487	5.490984	0.017682
hsa-miR-2861	5.871969	6.737994	5.993733	6.201232	7.520504	9.00987	7.603894	8.044756	1.843524
hsa-miR-299-3p	3.258541	2.951568	4.159781	3.45663	3.173158	2.839264	3.592279	3.201567	-0.25506
hsa-miR-299-5p	6.376744	5.882764	6.618713	6.29274	5.015507	4.217263	5.91885	5.05054	-1.2422
hsa-miR-29a-3p	10.92503	9.927593	11.7884	10.88034	11.01672	11.4896	11.97488	11.49373	0.613395
hsa-miR-29a-5p	2.809586	2.968667	2.994438	2.92423	2.905536	2.891823	3.037189	2.94485	0.020619
hsa-miR-29b-1-5p	4.962687	5.017355	5.51595	5.165331	4.20304	3.751634	3.9143	3.956324	-1.20901
hsa-miR-29b-3p	7.868356	7.0383	10.55583	8.487495	9.367001	9.53889	10.48716	9.797684	1.310188
hsa-miR-29c-3p	7.978154	7.199515	9.189611	8.122426	8.319091	8.756599	9.145971	8.740554	0.618127
hsa-miR-301a-3p	4.401829	3.563228	5.111365	4.358808	5.34108	4.637246	4.697313	4.89188	0.533072
hsa-miR-30a-3p	5.571334	5.068721	5.196894	5.278983	4.998527	5.34139	5.494272	5.278063	-0.00092
hsa-miR-30a-5p	8.408984	7.850891	7.94782	8.069232	8.226136	8.4749	8.643011	8.448015	0.378784
hsa-miR-30b-5p	6.378446	5.615494	6.80997	6.26797	6.588116	6.408088	6.681849	6.559351	0.291381
hsa-miR-30c-5p	5.756706	4.968919	5.757007	5.494211	5.728159	6.108326	5.98804	5.941508	0.447297
hsa-miR-30d-5p	6.304788	6.227754	5.812716	6.115086	6.040911	5.674155	5.896478	5.870514	-0.24457

hsa-miR-30e-3p	4.433933	4.075858	4.47613	4.328641	4.404931	4.398415	4.572973	4.458773	0.130132
hsa-miR-30e-5p	5.516098	5.028697	5.843091	5.462628	6.185722	6.217527	6.27352	6.22559	0.762961
hsa-miR-3125	5.014794	5.634033	4.359359	5.002729	4.118414	3.977536	4.045758	4.047236	-0.95549
hsa-miR-3127-5p	4.148207	5.124403	3.832919	4.36851	3.739893	3.621352	3.600681	3.653975	-0.71453
hsa-miR-3132	3.551447	2.906596	3.76367	3.407238	3.642818	3.632494	3.581324	3.618879	0.211641
hsa-miR-31-3p	6.428833	6.237328	8.004286	6.890149	7.135415	6.801506	7.638467	7.191796	0.301647
hsa-miR-3141	3.396819	4.256752	3.411908	3.688493	3.67698	4.115877	3.658219	3.817026	0.128533
hsa-miR-3156-5p	3.931649	4.870233	3.711688	4.17119	3.568363	3.598252	3.615762	3.594126	-0.57706
hsa-miR-31-5p	7.847898	7.836741	8.679573	8.121404	7.862847	7.716976	8.428577	8.0028	-0.1186
hsa-miR-3162-5p	8.010217	7.967143	7.749062	7.908808	9.383209	11.38969	9.361494	10.0448	2.135992
hsa-miR-3188	2.993478	3.100954	3.345956	3.146796	5.382878	6.404365	5.05274	5.613328	2.466532
hsa-miR-3195	6.713852	7.103149	6.603405	6.806802	7.782585	9.251494	7.917444	8.317174	1.510372
hsa-miR-3196	5.98895	6.030784	6.183622	6.067786	7.244567	8.625457	7.545991	7.805338	1.737553
hsa-miR-3198	6.414878	7.016184	5.905383	6.445482	5.43837	5.510915	5.322951	5.424079	-1.0214
hsa-miR-320a	6.634567	7.037449	6.347591	6.673202	5.749211	5.93943	5.92897	5.872537	-0.80067
hsa-miR-320b	7.717215	8.23056	7.500412	7.816062	6.862013	7.040615	7.170472	7.024367	-0.7917
hsa-miR-320c	7.007354	7.537122	6.772453	7.105643	6.457265	6.602637	6.501919	6.520607	-0.58504
hsa-miR-320d	8.250456	8.846676	7.956072	8.351068	7.411202	7.655935	7.763204	7.610113	-0.74096
hsa-miR-320e	7.77761	8.439819	7.517217	7.911549	6.859998	7.119185	7.272538	7.083907	-0.82764
hsa-miR-324-3p	7.414341	7.654056	6.915525	7.327974	6.55533	6.249171	6.452011	6.418837	-0.90914
hsa-miR-324-5p	6.240469	5.857998	5.944058	6.014175	6.227516	6.063433	5.878037	6.056329	0.042154
hsa-miR-329	3.881961	3.745538	3.729053	3.785517	3.289616	3.211298	3.638267	3.379727	-0.40579
hsa-miR-331-3p	7.625232	7.601528	7.195461	7.474074	7.442912	7.225534	7.247641	7.305362	-0.16871
hsa-miR-335-5p	4.953249	4.723456	5.253049	4.976585	5.868608	4.797387	4.677978	5.114658	0.138073
hsa-miR-337-3p	4.060637	3.495417	4.967197	4.174417	4.084657	3.928151	4.873951	4.295586	0.12117
hsa-miR-337-5p	5.553527	4.74971	6.354816	5.552684	4.972581	4.793686	6.215389	5.327219	-0.22547
hsa-miR-342-3p	6.115923	6.488867	5.75358	6.119456	5.909729	5.751474	5.88052	5.847241	-0.27222
hsa-miR-34a-3p	2.877087	2.829888	3.195962	2.967646	3.279514	3.317647	3.423514	3.340225	0.372579
hsa-miR-34a-5p	9.164315	9.392581	8.911347	9.156081	9.893155	9.871642	10.1933	9.986032	0.82995
hsa-miR-34b-5p	5.737252	6.143558	5.637864	5.839558	6.053933	6.307168	6.638273	6.333125	0.493567
hsa-miR-34c-5p	3.49371	3.314329	3.998291	3.60211	3.546495	3.696206	4.179308	3.807336	0.205227
hsa-miR-361-3p	3.918391	3.716348	3.879296	3.838012	3.68007	3.650033	3.69219	3.674097	-0.16391
hsa-miR-361-5p	7.217552	7.66302	6.631329	7.170634	6.498474	6.72841	6.612392	6.613092	-0.55754
hsa-miR-362-3p	2.856142	2.779852	3.666444	3.100812	3.828659	3.802419	4.108258	3.913112	0.8123
hsa-miR-362-5p	3.792535	3.695482	3.881174	3.78973	3.946143	3.954777	3.907057	3.935992	0.146262
hsa-miR-3648	3.112296	3.015173	3.6993	3.27559	4.565503	5.829566	4.921938	5.105669	1.830079
hsa-miR-3651	7.208448	7.956121	5.984392	7.049654	6.379993	5.681218	5.096994	5.719402	-1.33025

hsa-miR-3652	3.740356	4.099506	3.474506	3.771456	4.277575	4.856245	3.955359	4.36306	0.591604
hsa-miR-3653	3.620989	3.498833	3.491122	3.536981	4.00786	3.872898	3.620429	3.833729	0.296748
hsa-miR-3656	4.727644	5.929102	4.669973	5.108907	6.118941	7.110261	6.083485	6.437563	1.328656
hsa-miR-3659	3.030038	2.758017	3.574645	3.1209	3.481263	3.474943	3.450678	3.468961	0.348062
hsa-miR-365a-3p	10.78795	10.38469	10.4361	10.53625	10.25543	10.30748	10.37437	10.31243	-0.22382
hsa-miR-3663-3p	4.330718	4.50162	6.24001	5.024116	8.312613	7.979441	8.28826	8.193438	3.169322
hsa-miR-3665	7.798442	8.345297	8.22389	8.122543	9.979671	10.43563	10.0191	10.1448	2.022257
hsa-miR-3679-5p	4.798294	5.520577	4.748688	5.02252	5.165952	5.579423	4.911985	5.21912	0.1966
hsa-miR-3682-3p	2.843116	2.888621	2.844828	2.858855	3.503073	4.945528	3.483419	3.97734	1.118485
hsa-miR-369-5p	3.835185	3.313834	4.122377	3.757132	3.646352	3.5127	4.170187	3.776413	0.019281
hsa-miR-370	3.955929	3.901613	3.557668	3.80507	3.398973	3.46056	3.38082	3.413451	-0.39162
hsa-miR-371a-5p	4.201174	4.153898	4.201854	4.185642	5.574464	6.640742	5.891524	6.035577	1.849935
hsa-miR-373-5p	2.716516	2.853792	2.96356	2.844623	3.259827	3.54736	3.462622	3.42327	0.578647
hsa-miR-374a-5p	6.076569	4.900551	7.13459	6.037237	6.841182	6.890366	7.109319	6.946956	0.909719
hsa-miR-374b-5p	5.738416	5.080558	5.629122	5.482698	5.396577	5.443991	5.368053	5.402874	-0.07982
hsa-miR-376a-3p	6.882407	6.334495	7.986263	7.067722	6.636434	6.718596	7.862168	7.072399	0.004677
hsa-miR-376a-5p	4.14481	3.598314	4.7736	4.172241	4.196273	4.007786	4.80818	4.337413	0.165172
hsa-miR-376b	3.685733	3.132788	5.069573	3.962698	3.707976	3.527387	4.509434	3.914932	-0.04777
hsa-miR-376c	7.427112	6.906287	8.278419	7.537273	7.032398	6.89783	8.069611	7.333279	-0.20399
hsa-miR-377-3p	6.260538	5.520422	7.73496	6.505307	6.449863	6.190408	7.886976	6.842416	0.337109
hsa-miR-377-5p	3.110088	3.37189	3.084764	3.188914	2.867392	2.859297	3.084835	2.937175	-0.25174
hsa-miR-379-5p	6.303023	6.186065	6.350568	6.279885	5.059815	4.455073	5.815137	5.110008	-1.16988
hsa-miR-381	4.670836	3.837949	5.916554	4.808447	4.191684	3.758654	5.099944	4.350094	-0.45835
hsa-miR-382-5p	5.576084	5.982829	4.98337	5.514094	4.239521	4.163339	4.77498	4.392613	-1.12148
hsa-miR-3911	2.941035	2.943021	3.539185	3.14108	3.642892	3.751492	3.577931	3.657438	0.516358
hsa-miR-3937	2.930519	3.025076	3.131062	3.028886	3.676898	4.016272	3.704126	3.799099	0.770213
hsa-miR-409-3p	6.823709	7.089018	6.442668	6.785132	5.107057	5.010021	6.299088	5.472055	-1.31308
hsa-miR-409-5p	4.73748	4.567411	4.414099	4.572997	3.941301	3.866219	4.521772	4.109764	-0.46323
hsa-miR-410	5.399112	4.991398	5.394191	5.261567	4.711379	4.542449	5.07507	4.776299	-0.48527
hsa-miR-411-5p	3.400838	2.866772	4.622358	3.62999	3.643835	3.158211	4.278356	3.693467	0.063478
hsa-miR-423-5p	4.569999	4.491547	4.215083	4.425543	4.159652	4.1034	4.008689	4.09058	-0.33496
hsa-miR-424-5p	7.572502	7.247288	8.537791	7.78586	7.466249	8.534217	8.183301	8.061255	0.275395
hsa-miR-425-5p	5.299914	4.461092	4.998421	4.919809	4.951632	4.298064	4.466395	4.57203	-0.34778
hsa-miR-4257	3.59675	4.125329	3.527306	3.749795	4.313687	6.151636	4.875483	5.113602	1.363807
hsa-miR-4270	3.514776	4.005079	3.914083	3.811313	5.136398	6.008092	4.874583	5.339691	1.528378
hsa-miR-4271	5.292838	5.935213	4.962254	5.396768	6.145593	6.494709	6.178708	6.273004	0.876235
hsa-miR-4281	7.885258	8.245039	9.143329	8.424542	10.64416	10.45542	10.43161	10.5104	2.085855

hsa-miR-4284	10.22322	10.48661	10.65617	10.45533	10.00354	10.27073	9.754555	10.00961	-0.44573
hsa-miR-4286	9.845686	9.193282	10.33796	9.79231	10.1112	9.750982	9.845906	9.902695	0.110385
hsa-miR-4291	5.070843	4.2366	4.793772	4.700405	4.885542	5.019143	4.915932	4.940206	0.239801
hsa-miR-4298	3.112935	4.401858	3.379219	3.631338	3.67285	4.141081	3.697705	3.837212	0.205875
hsa-miR-4299	7.200719	7.328346	6.642985	7.05735	6.445845	6.755571	6.724576	6.641998	-0.41535
hsa-miR-4306	6.101251	5.638874	5.819254	5.853127	5.962658	5.99703	5.772521	5.910736	0.05761
hsa-miR-4313	3.657405	4.009093	3.470129	3.712209	3.283573	3.074413	3.501964	3.28665	-0.42556
hsa-miR-431-5p	3.676351	3.142064	4.005626	3.608014	3.52004	3.438132	3.780611	3.579594	-0.02842
hsa-miR-4317	4.194822	4.274963	3.983629	4.151138	4.03048	4.03796	4.003108	4.023849	-0.12729
hsa-miR-4324	4.226454	4.065854	3.901534	4.064614	4.095643	4.05441	3.955433	4.035162	-0.02945
hsa-miR-432-5p	5.42276	6.227221	4.691675	5.447219	4.335982	4.341504	4.667861	4.448449	-0.99877
hsa-miR-4327	4.957632	5.390611	4.92302	5.090421	6.364076	6.593861	6.397063	6.451667	1.361246
hsa-miR-450a-5p	3.895629	3.349339	4.642746	3.962571	4.075548	4.492008	3.986932	4.184829	0.222258
hsa-miR-454-3p	3.929354	3.514454	3.555886	3.666565	3.611985	3.475251	3.417324	3.50152	-0.16504
hsa-miR-455-3p	6.830543	7.265802	6.580784	6.892376	6.477356	6.065304	6.097521	6.213394	-0.67898
hsa-miR-483-5p	2.954947	3.332988	3.262189	3.183375	3.562244	3.47827	3.369294	3.469936	0.286561
hsa-miR-484	4.701568	4.31837	4.472969	4.497635	4.235596	3.831267	3.917738	3.994867	-0.50277
hsa-miR-485-3p	4.174838	4.744648	3.958066	4.292518	3.22936	3.202734	3.874795	3.43563	-0.85689
hsa-miR-487b	6.064277	5.798585	5.861909	5.908257	5.201479	4.938169	5.56679	5.235479	-0.67278
hsa-miR-493-5p	7.054671	7.203449	6.548066	6.935395	5.63891	5.450485	6.366056	5.818484	-1.11691
hsa-miR-494	8.319163	8.961802	9.161459	8.814141	8.980543	8.888252	9.514362	9.127719	0.313578
hsa-miR-495	5.445382	5.167168	5.729965	5.447505	4.723282	4.784269	5.697013	5.068188	-0.37932
hsa-miR-497-5p	4.937724	4.203246	4.950667	4.697212	4.687182	4.730151	5.075125	4.830819	0.133607
hsa-miR-498	3.524212	3.804917	3.432086	3.587072	3.723546	3.747207	3.612222	3.694325	0.107253
hsa-miR-500a-3p	3.306468	3.241469	3.407529	3.318489	3.581748	3.518728	3.634688	3.578388	0.259899
hsa-miR-501-5p	3.274793	3.556612	3.084079	3.305161	3.183692	3.104792	3.218448	3.168977	-0.13618
hsa-miR-502-3p	3.138104	3.027351	3.369662	3.178372	3.489258	3.331636	3.492282	3.437725	0.259353
hsa-miR-503	3.402776	2.875704	3.664522	3.314334	3.217895	3.423789	3.273937	3.305207	-0.00913
hsa-miR-505-3p	5.166049	5.149438	4.856736	5.057407	4.623083	4.071502	4.565921	4.420169	-0.63724
hsa-miR-505-5p	3.701921	3.722728	3.25785	3.560833	3.349047	3.151161	3.215478	3.238562	-0.32227
hsa-miR-513a-5p	3.740252	3.837073	3.885071	3.820799	4.024973	4.427073	3.79222	4.081422	0.260623
hsa-miR-513b	2.952346	3.072425	3.037009	3.020593	3.236996	3.368383	3.215821	3.273733	0.25314
hsa-miR-532-3p	3.567659	3.92654	3.484515	3.659571	3.607123	3.452848	3.509614	3.523195	-0.13638
hsa-miR-532-5p	4.108689	4.146523	3.967516	4.074242	4.167518	4.053078	4.051498	4.090698	0.016456
hsa-miR-539-5p	4.297989	4.395353	3.642741	4.112028	3.49763	3.411932	3.615762	3.508441	-0.60359
hsa-miR-542-3p	3.817393	3.41637	4.343371	3.859045	4.141005	4.496211	4.037372	4.224862	0.365818
hsa-miR-542-5p	4.25305	4.422782	3.894798	4.19021	3.733986	4.054212	3.671751	3.819983	-0.37023

hsa-miR-543	4.661613	5.12933	4.789599	4.860181	3.785496	3.557506	4.382953	3.908652	-0.95153
hsa-miR-551b-3p	6.13275	4.097929	5.179246	5.136642	8.395185	5.512223	5.182129	6.363179	1.226537
hsa-miR-572	3.377587	3.619602	3.414622	3.470604	3.894319	4.984757	4.158182	4.345752	0.875149
hsa-miR-574-3p	6.209303	7.025217	5.700051	6.311524	5.534554	5.641864	5.55707	5.57783	-0.73369
hsa-miR-574-5p	6.374362	6.688203	5.887039	6.316535	5.627009	5.398765	5.409854	5.478542	-0.83799
hsa-miR-575	5.687476	6.082969	5.111434	5.627293	5.567059	5.34998	4.722874	5.213305	-0.41399
hsa-miR-590-5p	3.64237	3.031236	5.08539	3.919665	4.474726	4.339281	4.563878	4.459295	0.53963
hsa-miR-615-3p	2.842044	2.815487	2.73136	2.796297	2.677296	2.722317	2.82353	2.741048	-0.05525
hsa-miR-625-5p	3.591929	3.460538	3.460071	3.504179	3.149006	2.93235	3.111526	3.064294	-0.43989
hsa-miR-629-3p	2.87956	2.960124	3.184204	3.007963	3.500795	4.863975	4.169664	4.178145	1.170182
hsa-miR-629-5p	2.812116	2.957657	2.800805	2.856859	2.69072	2.5672	2.580162	2.612694	-0.24417
hsa-miR-630	5.89258	6.452603	6.772205	6.372463	7.202182	5.897114	6.506365	6.53522	0.162757
hsa-miR-636	3.017092	3.10132	2.875163	2.997858	3.362885	3.70635	3.459122	3.509453	0.511594
hsa-miR-638	5.98997	6.860844	6.213492	6.354768	7.180201	8.944589	7.530589	7.885126	1.530358
hsa-miR-642b-3p	4.234645	4.372931	4.644513	4.417363	6.17054	7.212993	6.387788	6.59044	2.173078
hsa-miR-654-3p	5.6666	5.706102	6.240367	5.871023	5.049815	4.944435	6.096605	5.363618	-0.5074
hsa-miR-660-5p	3.365731	3.178244	4.274206	3.60606	4.600312	4.58733	4.746025	4.644556	1.038495
hsa-miR-663a	2.824646	2.949648	3.215855	2.996716	4.107604	4.651848	4.070279	4.276577	1.279861
hsa-miR-664-3p	3.626433	3.647312	3.462203	3.578649	3.836536	3.935033	3.758217	3.843262	0.264613
hsa-miR-671-5p	2.710773	3.396243	3.269641	3.125552	3.468335	3.788625	3.465798	3.574253	0.4487
hsa-miR-718	2.739418	2.643172	3.093983	2.825524	3.779791	3.878528	3.782364	3.813561	0.988037
hsa-miR-720	14.19764	13.90475	14.1896	14.09733	14.06189	13.61803	13.8842	13.85471	-0.24262
hsa-miR-744-5p	3.637866	3.817744	3.613423	3.689678	3.578973	3.374258	3.526973	3.493401	-0.19628
hsa-miR-758	4.858394	4.74327	4.74033	4.780665	3.797776	3.650528	4.220039	3.889448	-0.89122
hsa-miR-7-5p	3.983932	3.450357	4.592071	4.008787	3.735499	3.30242	3.408969	3.482296	-0.52649
hsa-miR-762	6.458658	6.281371	6.420134	6.386721	9.544479	10.41481	9.24774	9.735678	3.348957
hsa-miR-769-5p	3.370388	3.187346	3.54361	3.367115	3.403246	3.294063	3.359243	3.352184	-0.01493
hsa-miR-874	4.661553	4.426192	4.463624	4.517123	6.035447	7.42779	6.17323	6.545489	2.028366
hsa-miR-887	2.667496	2.641188	2.685865	2.66485	3.0421	3.446948	3.214579	3.234542	0.569693
hsa-miR-892b	3.975173	4.439126	3.569548	3.994616	3.485535	3.386821	3.444831	3.439062	-0.55555
hsa-miR-92a-3p	7.409589	7.673444	6.957163	7.346732	6.302609	5.073023	5.459489	5.611707	-1.73502
hsa-miR-93-5p	6.576028	5.977112	6.273155	6.275432	5.767192	5.157281	5.010105	5.311526	-0.96391
hsa-miR-939	5.305455	5.213142	5.056632	5.191743	5.829321	6.579419	5.516201	5.97498	0.783237
hsa-miR-940	5.828426	6.08433	5.57778	5.830179	6.330771	7.752004	6.787686	6.956821	1.126642
hsa-miR-98	6.608955	6.620803	5.959067	6.396275	6.247716	6.143794	5.776644	6.056052	-0.34022
hsa-miR-99a-5p	6.823914	6.685189	7.422422	6.977175	6.761119	6.918567	7.590129	7.089938	0.112763
hsa-miR-99b-5p	7.758907	7.730682	6.89685	7.462146	6.931624	6.927617	6.758678	6.87264	-0.58951

				old donors				
miRNA	164_4	194_4	271_4	average	164_8	194_8	271_8	average	FC o8-o4
hsa-let-7a-3p	2.748402	2.672562	2.824938	2.748634	2.767893	2.667083	2.741869	2.725615	-0.02302
hsa-let-7a-5p	12.9111	13.38129	13.03326	13.10855	13.43652	13.62665	13.36236	13.47517	0.366625
hsa-let-7b-5p	12.12615	12.42855	12.02608	12.1936	12.56948	12.50137	12.43022	12.50036	0.30676
hsa-let-7c	10.44085	10.53917	10.19023	10.39008	10.6629	10.56192	11.0938	10.77288	0.382794
hsa-let-7d-5p	9.0194	9.344247	9.317699	9.227115	9.20297	9.224462	9.639905	9.355779	0.128664
hsa-let-7e-5p	10.22886	10.41475	10.12175	10.25512	10.60719	10.65531	11.00062	10.75438	0.499259
hsa-let-7f-5p	12.2068	12.34743	12.34279	12.29901	12.4065	12.54888	12.50746	12.48761	0.188609
hsa-let-7g-5p	9.679509	10.14996	9.812185	9.880552	10.02536	9.668598	10.00028	9.898082	0.01753
hsa-let-7i-5p	11.053	12.25245	11.37215	11.5592	11.80135	11.60953	12.02225	11.81104	0.251844
hsa-miR-100-5p	11.14144	11.24212	11.91652	11.43336	11.13904	11.46308	11.73245	11.44486	0.011494
hsa-miR-101-3p	3.995757	5.78768	4.512646	4.765361	4.693739	4.725994	3.283682	4.234472	-0.53089
hsa-miR-103a-3p	8.954096	9.27975	8.96106	9.064969	9.448804	9.340171	9.099223	9.296066	0.231097
hsa-miR-106b-5p	6.92629	7.231568	7.85516	7.337673	5.606861	5.622456	6.119255	5.782858	-1.55481
hsa-miR-107	8.942622	9.244356	8.886283	9.02442	9.156138	9.123871	9.018633	9.099547	0.075127
hsa-miR-10a-5p	4.011895	5.774091	5.931444	5.239143	4.387631	4.73603	6.091525	5.071729	-0.16741
hsa-miR-10b-5p	7.716794	8.107112	7.424763	7.749556	8.248481	7.784305	7.759457	7.930748	0.181191
hsa-miR-1181	3.345506	4.976083	3.542407	3.954665	6.150836	7.346313	4.26753	5.92156	1.966894
hsa-miR-1185-5p	3.602148	3.569671	3.298197	3.490005	3.64164	3.346698	3.236306	3.408214	-0.08179
hsa-miR-1202	6.347946	5.959318	6.314195	6.207153	6.502625	7.787274	6.561437	6.950445	0.743292
hsa-miR-1207-5p	6.325882	7.019575	6.090709	6.478722	8.348629	9.241019	8.084293	8.55798	2.079258
hsa-miR-1224-5p	3.088323	3.222539	2.956934	3.089266	3.14069	5.731366	3.069396	3.980484	0.891218
hsa-miR-1225-3p	3.745695	3.483456	3.608709	3.61262	3.290386	4.223922	3.898669	3.804326	0.191705
hsa-miR-1225-5p	6.464108	6.61101	6.123151	6.399423	7.687673	8.548781	7.019965	7.75214	1.352717
hsa-miR-1226-5p	2.841573	3.083385	2.761875	2.895611	3.155067	4.175055	3.098461	3.476194	0.580583
hsa-miR-1228-3p	4.465546	3.901534	4.156068	4.174383	3.095591	4.007401	4.838263	3.980418	-0.19396
hsa-miR-1234	4.653481	4.199783	4.422561	4.425275	4.519161	5.455073	5.310688	5.094974	0.669699
hsa-miR-1238	3.856976	3.567043	3.79786	3.740626	3.071138	3.382075	4.053294	3.502169	-0.23846
hsa-miR-1246	3.835679	3.934547	4.056619	3.942282	2.985706	4.029366	4.871968	3.962347	0.020065
hsa-miR-1249	2.971279	2.914043	2.966636	2.950652	2.884846	3.516114	2.89477	3.098577	0.147924
hsa-miR-125a-3p	3.768618	3.9799	3.804949	3.851156	3.413368	5.128192	3.806265	4.115942	0.264786
hsa-miR-125a-5p	8.157414	8.047234	8.039552	8.0814	8.014615	7.784794	8.538854	8.112754	0.031355
hsa-miR-125b-5p	12.64548	12.97126	12.50743	12.70806	12.92328	13.1673	12.66289	12.91782	0.209768
hsa-miR-1260a	10.16779	9.23609	10.19124	9.865041	9.132888	8.592364	10.03384	9.25303	-0.61201
hsa-miR-1260b	9.869865	9.14436	9.967007	9.660411	8.57879	8.436243	9.844953	8.953329	-0.70708
hsa-miR-1268a	4.147135	3.860889	3.962185	3.99007	4.70277	5.449055	4.388395	4.84674	0.85667

hsa-miR-1271-5p	3.98883	3.877701	4.227694	4.031409	3.532912	3.161655	3.636957	3.443842	-0.58757
hsa-miR-127-3p	8.053518	7.46668	7.310439	7.610212	8.385884	7.597791	8.421658	8.135111	0.524899
hsa-miR-1274a_v16	10.07921	8.765524	9.55433	9.466355	8.934266	8.569647	9.238461	8.914125	-0.55223
hsa-miR-1274b_v16	12.51332	11.78387	12.66655	12.32125	11.516	11.1873	11.98084	11.56138	-0.75987
hsa-miR-1275	4.822498	4.104009	5.252872	4.72646	3.786549	5.287624	5.316118	4.796764	0.070304
hsa-miR-128	4.726665	5.226155	5.195588	5.049469	4.332986	4.74448	5.1179	4.731789	-0.31768
hsa-miR-1280	6.476989	5.591717	6.323117	6.130608	5.50022	5.620243	6.436018	5.85216	-0.27845
hsa-miR-1288	4.41515	4.154431	4.422561	4.330714	3.911114	3.991786	5.389388	4.430763	0.100049
hsa-miR-1290	3.44562	3.499362	3.183482	3.376155	2.690871	3.928902	4.227958	3.61591	0.239756
hsa-miR-1305	6.373121	5.914529	6.15925	6.148966	5.093025	5.558871	6.920496	5.857464	-0.2915
hsa-miR-130a-3p	9.467381	9.796858	9.974108	9.746116	9.42903	9.071508	9.609782	9.370107	-0.37601
hsa-miR-130b-3p	6.958193	6.35769	7.340654	6.885512	5.246435	5.570721	6.595176	5.80411	-1.0814
hsa-miR-132-3p	6.078684	5.635422	4.664243	5.45945	4.394975	4.153416	5.550881	4.699757	-0.75969
hsa-miR-132-5p	3.670916	3.753592	3.286743	3.570417	3.39982	3.134114	3.363171	3.299035	-0.27138
hsa-miR-134	4.74419	4.780051	4.36833	4.630857	5.944762	6.68128	5.973581	6.199874	1.569017
hsa-miR-135a-3p	2.904296	3.144243	2.948259	2.998933	2.886934	4.5494	3.119757	3.518697	0.519764
hsa-miR-136-3p	4.144359	4.398663	3.979315	4.174113	4.16025	3.737945	2.901935	3.600043	-0.57407
hsa-miR-136-5p	5.181045	5.187377	5.373602	5.247341	5.673844	4.372149	3.419223	4.488405	-0.75894
hsa-miR-137	7.383209	7.766243	6.471203	7.206885	8.069917	8.310287	5.995837	7.45868	0.251795
hsa-miR-138-5p	4.88799	3.952387	4.895601	4.578659	2.809422	3.200388	4.037487	3.349099	-1.22956
hsa-miR-140-3p	7.791713	8.293046	7.904672	7.996477	8.092577	8.182217	7.648539	7.974444	-0.02203
hsa-miR-140-5p	8.503604	9.406693	8.735047	8.881781	9.14747	8.882919	7.803093	8.611161	-0.27062
hsa-miR-143-3p	6.113762	6.71479	6.113461	6.314004	6.010902	6.22278	5.320998	5.85156	-0.46244
hsa-miR-143-5p	4.000324	4.472641	3.768842	4.080602	3.81099	3.98944	3.80763	3.869353	-0.21125
hsa-miR-145-3p	4.647442	4.884204	5.023642	4.851763	4.23639	4.348737	4.224661	4.26993	-0.58183
hsa-miR-145-5p	9.608824	9.73081	9.355854	9.565163	9.87834	9.97545	9.880968	9.911586	0.346423
hsa-miR-146b-5p	3.029097	3.373012	4.402995	3.601702	2.762958	2.92132	3.963184	3.215821	-0.38588
hsa-miR-148a-3p	6.553159	8.077114	7.061678	7.23065	5.981096	6.253673	5.241011	5.82526	-1.40539
hsa-miR-148b-3p	4.534759	5.307107	4.93401	4.925292	4.834617	4.784449	4.112814	4.577293	-0.348
hsa-miR-149-5p	3.930539	3.825152	4.031525	3.929072	4.535398	3.884535	3.734013	4.051315	0.122243
hsa-miR-150-3p	4.021029	5.502813	3.942404	4.488749	6.53842	7.549091	5.418479	6.501997	2.013248
hsa-miR-151a-3p	5.624245	5.687064	5.689755	5.667021	5.147415	4.939754	5.646081	5.244417	-0.4226
hsa-miR-151a-5p	7.435841	7.889303	7.298028	7.541057	7.751636	7.499142	7.510909	7.587229	0.046172
hsa-miR-152	5.658222	6.408668	5.576928	5.881273	6.022091	6.220769	5.215244	5.819368	-0.0619
hsa-miR-154-3p	6.104339	4.86974	5.837254	5.603778	5.165709	3.819491	4.618656	4.534619	-1.06916
hsa-miR-154-5p	4.266618	4.322667	4.124537	4.237941	5.005248	4.018529	4.125924	4.383234	0.145293
hsa-miR-155-5p	5.032132	4.066804	5.751093	4.95001	2.68847	2.986611	5.886653	3.853911	-1.0961

hsa-miR-15a-5p	8.08534	8.647269	8.57609	8.436233	7.880436	7.434007	7.385069	7.566504	-0.86973
hsa-miR-15b-5p	9.757689	9.223612	10.21144	9.730913	8.222162	7.87747	9.081436	8.39369	-1.33722
hsa-miR-16-2-3p	2.745065	2.939607	3.048356	2.911009	2.84969	2.612631	2.716111	2.726144	-0.18487
hsa-miR-16-5p	10.50066	10.3456	10.83109	10.55912	9.921892	9.384313	10.17563	9.827278	-0.73184
hsa-miR-17-5p	7.011014	6.811665	7.77713	7.199936	5.113141	4.533334	6.631506	5.425994	-1.77394
hsa-miR-181a-2-3p	2.880195	3.046253	3.235676	3.054041	3.683762	3.205885	3.155695	3.348447	0.294406
hsa-miR-181a-3p	3.258683	3.655553	3.577716	3.497317	4.034047	3.629017	3.100876	3.58798	0.090663
hsa-miR-181a-5p	6.258066	7.631666	6.194031	6.694588	8.65899	8.517986	7.853089	8.343355	1.648767
hsa-miR-181b-5p	5.085215	5.410349	5.473481	5.323015	6.199958	5.675433	6.182743	6.019378	0.696363
hsa-miR-181c-5p	2.856926	3.630649	2.954996	3.147524	3.612398	3.804888	3.175954	3.53108	0.383557
hsa-miR-181d	3.019913	3.467594	3.120248	3.202585	3.666532	3.458377	3.266593	3.463834	0.261249
hsa-miR-185-5p	5.603992	5.96966	5.957127	5.843593	5.070109	5.726683	4.788975	5.195255	-0.64834
hsa-miR-186-5p	4.144944	4.915665	4.057352	4.372654	4.091165	4.466669	4.078373	4.212069	-0.16058
hsa-miR-187-5p	2.55966	2.777895	2.644609	2.660721	2.916371	4.450107	2.666646	3.344375	0.683653
hsa-miR-188-5p	4.579812	4.032997	4.101769	4.238193	3.360015	4.939785	4.203891	4.167897	-0.0703
hsa-miR-18a-5p	4.389803	3.826029	5.384754	4.533529	2.687244	2.876163	3.069137	2.877514	-1.65601
hsa-miR-18b-5p	2.954995	3.017203	3.520736	3.164311	2.743211	2.721535	2.800944	2.75523	-0.40908
hsa-miR-191-3p	3.904825	3.662765	3.955407	3.840999	2.791979	3.35519	3.923075	3.356748	-0.48425
hsa-miR-1914-3p	4.822355	4.246052	4.784662	4.61769	3.74977	4.334173	5.619954	4.567966	-0.04972
hsa-miR-1915-3p	6.206433	7.102914	6.122061	6.477136	8.662167	10.376	7.183121	8.740431	2.263295
hsa-miR-193a-3p	6.146081	6.515051	6.701978	6.45437	6.044333	5.523137	4.768523	5.445331	-1.00904
hsa-miR-193a-5p	6.307237	5.91174	6.134303	6.11776	6.073151	6.014141	6.922133	6.336475	0.218715
hsa-miR-193b-3p	7.81393	8.061586	7.6571	7.844205	7.679177	7.289722	7.773	7.580633	-0.26357
hsa-miR-193b-5p	3.357701	3.449375	3.377176	3.394751	2.779919	3.414883	3.584905	3.259902	-0.13485
hsa-miR-195-5p	6.785114	7.691364	7.06408	7.180186	7.106808	6.63157	6.427116	6.721831	-0.45835
hsa-miR-196a-5p	6.492383	7.613949	8.036215	7.380849	7.028513	7.437104	8.649331	7.704983	0.324134
hsa-miR-196b-5p	6.913229	6.704903	7.014478	6.877536	6.682908	6.224948	7.278885	6.728914	-0.14862
hsa-miR-1973	4.32338	4.173532	4.230684	4.242532	3.208135	4.250212	4.675099	4.044482	-0.19805
hsa-miR-197-3p	4.205274	4.172204	4.198722	4.192067	5.76705	4.225034	4.336125	4.77607	0.584003
hsa-miR-199a-3p	11.33874	11.94753	11.7165	11.66759	11.74895	11.42181	11.21142	11.46072	-0.20687
hsa-miR-199a-5p	8.926621	9.907488	9.471819	9.435309	9.66558	9.027331	8.306076	8.999662	-0.43565
hsa-miR-199b-5p	3.497746	3.860285	4.533052	3.963694	3.761971	2.798686	2.823018	3.127891	-0.8358
hsa-miR-19a-3p	6.98663	7.143173	8.07413	7.401311	5.199138	4.548334	5.70581	5.151094	-2.25022
hsa-miR-19b-3p	8.353128	8.833459	9.392077	8.859555	7.478085	6.474491	7.674746	7.209107	-1.65045
hsa-miR-20a-5p	7.803514	7.742054	8.558462	8.034677	5.931523	5.294753	7.46342	6.229899	-1.80478
hsa-miR-20b-5p	5.106971	5.168633	5.951208	5.408937	3.648116	3.525949	4.765798	3.979954	-1.42898
hsa-miR-210	6.34067	6.568697	4.556321	5.821896	6.28759	5.775348	6.606209	6.223049	0.401153

hsa-miR-21-3p	6.652365	6.339168	7.029356	6.67363	5.193292	5.103061	6.082194	5.459516	-1.21411
hsa-miR-214-3p	8.922579	9.706294	9.317538	9.31547	9.385335	8.952823	9.322234	9.220131	-0.09534
hsa-miR-214-5p	4.066358	4.767052	4.686386	4.506599	3.862832	3.607778	3.451908	3.640839	-0.86576
hsa-miR-21-5p	14.08735	14.83021	14.52541	14.48099	14.50816	13.93308	13.14492	13.86205	-0.61894
hsa-miR-218-5p	4.705691	4.058273	5.397384	4.720449	3.009487	3.181408	3.313372	3.168089	-1.55236
hsa-miR-221-3p	11.37124	10.78524	10.91574	11.02407	10.64343	10.42349	10.80413	10.62368	-0.40039
hsa-miR-221-5p	7.382384	6.990901	6.976374	7.116553	6.820395	6.139096	6.674609	6.5447	-0.57185
hsa-miR-222-3p	7.099559	7.1765	6.930995	7.069018	6.119201	5.997837	6.794863	6.303967	-0.76505
hsa-miR-22-3p	11.40578	11.79267	11.32671	11.50839	12.15992	11.89923	11.4722	11.84378	0.335396
hsa-miR-224-5p	6.063559	6.972052	7.07615	6.70392	3.408546	3.978736	7.574282	4.987188	-1.71673
hsa-miR-22-5p	5.778421	5.530091	5.861825	5.723446	5.453526	5.479596	4.999675	5.310932	-0.41251
hsa-miR-23a-3p	11.89292	12.67204	11.77841	12.11446	12.84817	12.87411	11.96479	12.56236	0.447897
hsa-miR-23b-3p	9.994388	10.77152	9.679975	10.14863	11.58884	11.7455	10.54535	11.29323	1.144605
hsa-miR-23b-5p	2.881418	3.014001	2.875155	2.923525	2.838101	3.277026	2.912981	3.009369	0.085845
hsa-miR-24-1-5p	3.13273	3.614101	3.235416	3.327416	3.459874	3.794101	2.853887	3.369287	0.041872
hsa-miR-24-3p	11.53661	11.82754	11.14121	11.50179	12.29398	12.26532	11.27806	11.94578	0.443998
hsa-miR-25-3p	7.204706	7.081821	7.818961	7.368496	5.953834	5.890073	7.408636	6.417515	-0.95098
hsa-miR-26a-5p	8.882269	9.470687	8.908814	9.087257	9.164521	8.669586	8.959636	8.931248	-0.15601
hsa-miR-26b-5p	8.115652	8.875442	8.062807	8.3513	8.584397	8.174109	8.137098	8.298535	-0.05277
hsa-miR-27a-3p	10.99364	11.46968	10.95134	11.13822	11.43579	10.61955	9.63088	10.56207	-0.57614
hsa-miR-27b-3p	10.1147	10.54034	9.923871	10.19297	10.98084	10.76533	9.715592	10.48725	0.294285
hsa-miR-28-5p	5.599277	6.242488	6.077879	5.973215	5.134953	5.227805	5.227169	5.196642	-0.77657
hsa-miR-2861	5.982533	5.533857	5.483081	5.66649	6.956117	8.762779	6.553933	7.424276	1.757786
hsa-miR-299-3p	4.043679	3.504527	3.732023	3.760076	2.749775	2.951688	3.115232	2.938898	-0.82118
hsa-miR-299-5p	7.075738	6.034753	6.428542	6.513011	6.465869	5.281932	6.387057	6.044953	-0.46806
hsa-miR-29a-3p	11.92451	11.57574	11.87567	11.79198	11.19452	10.91223	10.83747	10.98141	-0.81057
hsa-miR-29a-5p	3.051208	3.104451	2.936685	3.030781	2.870295	2.86273	2.812833	2.848619	-0.18216
hsa-miR-29b-1-5p	5.816285	4.194778	5.204052	5.071705	3.058623	3.55375	4.541659	3.718011	-1.35369
hsa-miR-29b-3p	10.36822	9.54188	10.45974	10.12328	9.165016	9.155971	8.10671	8.809232	-1.31405
hsa-miR-29c-3p	8.853497	9.022412	8.839208	8.905039	8.480219	8.299226	7.856518	8.211987	-0.69305
hsa-miR-301a-3p	4.53393	5.379566	5.264834	5.059443	4.270723	4.542487	3.477038	4.096749	-0.96269
hsa-miR-30a-3p	5.342008	5.35887	5.177345	5.292741	4.330344	4.987144	5.048981	4.788823	-0.50392
hsa-miR-30a-5p	7.923269	8.724409	7.646198	8.097958	8.229286	8.396881	7.910725	8.178964	0.081006
hsa-miR-30b-5p	6.506549	7.084813	6.64523	6.745531	6.489506	5.960619	5.731726	6.060617	-0.68491
hsa-miR-30c-5p	5.731708	6.240082	5.778693	5.916828	5.685234	5.757052	5.112504	5.518263	-0.39856
hsa-miR-30d-5p	5.689957	6.498661	5.610226	5.932948	6.136713	5.500182	5.89343	5.843442	-0.08951
hsa-miR-30e-3p	4.505292	4.757459	4.676207	4.646319	3.827402	4.093435	3.867394	3.92941	-0.71691

hsa-miR-30e-5p	5.604145	6.784942	5.902646	6.097245	6.345114	5.942412	5.230498	5.839341	-0.2579
hsa-miR-3125	4.938227	4.492511	4.681207	4.703982	3.082519	4.188782	5.637174	4.302825	-0.40116
hsa-miR-3127-5p	4.034106	3.822742	3.914237	3.923695	2.808947	3.743261	4.773704	3.775304	-0.14839
hsa-miR-3132	3.79921	3.360344	3.742427	3.633994	2.633889	3.480432	3.139332	3.084551	-0.54944
hsa-miR-31-3p	7.89784	7.507109	7.647298	7.684082	6.255505	5.163574	6.007383	5.808821	-1.87526
hsa-miR-3141	3.452085	3.431705	3.264666	3.382818	2.759268	3.974914	3.886981	3.540388	0.157569
hsa-miR-3156-5p	3.937495	3.661005	3.719913	3.772804	2.840964	3.742486	4.498038	3.69383	-0.07897
hsa-miR-31-5p	8.609724	8.591159	8.295633	8.498839	7.408234	6.558891	7.616253	7.194459	-1.30438
hsa-miR-3162-5p	7.64315	7.27045	7.436399	7.45	7.949503	10.04293	8.174577	8.722337	1.272337
hsa-miR-3188	2.829394	2.965323	2.747052	2.847256	3.863916	6.157167	2.972367	4.33115	1.483894
hsa-miR-3195	6.40546	6.170062	6.343072	6.306198	6.747091	8.418438	6.748573	7.304701	0.998503
hsa-miR-3196	5.734826	5.558566	5.679036	5.657476	6.382204	8.054998	6.043705	6.826969	1.169493
hsa-miR-3198	6.334305	5.793102	6.098789	6.075399	5.082969	5.540476	6.940322	5.854589	-0.22081
hsa-miR-320a	6.594485	6.409771	6.606111	6.536789	6.070038	6.085978	6.903569	6.353195	-0.18359
hsa-miR-320b	7.770949	7.531383	7.66986	7.657397	7.419915	7.31849	7.998122	7.578842	-0.07855
hsa-miR-320c	6.791653	6.870967	6.780762	6.814461	6.532057	6.775136	7.250048	6.852414	0.037953
hsa-miR-320d	7.997197	8.098227	7.988407	8.027944	8.078763	7.745995	8.530781	8.118513	0.090569
hsa-miR-320e	7.544509	7.605583	7.544852	7.564981	7.525065	7.186844	8.005811	7.572573	0.007592
hsa-miR-324-3p	7.160366	6.805418	7.145415	7.037066	6.758808	6.406174	7.731601	6.965528	-0.07154
hsa-miR-324-5p	5.845555	6.315689	6.158011	6.106418	6.191678	6.164328	5.769204	6.041736	-0.06468
hsa-miR-329	4.209705	3.430009	3.95903	3.866248	4.031755	3.321775	3.796668	3.716733	-0.14952
hsa-miR-331-3p	7.475945	7.550976	7.134676	7.387199	7.879724	7.444261	7.207107	7.510364	0.123165
hsa-miR-335-5p	5.477679	6.054874	6.615871	6.049475	2.892427	5.527436	5.237025	4.552296	-1.49718
hsa-miR-337-3p	4.843458	4.750825	4.662321	4.752201	5.599612	4.135831	3.891011	4.542151	-0.21005
hsa-miR-337-5p	6.370036	6.181937	6.098058	6.216677	6.479138	5.455366	5.469791	5.801432	-0.41525
hsa-miR-342-3p	5.542474	5.849519	5.806995	5.732996	5.970137	5.543776	6.390401	5.968105	0.235109
hsa-miR-34a-3p	3.111311	3.706065	3.206569	3.341315	3.331821	3.502272	3.043881	3.292658	-0.04866
hsa-miR-34a-5p	9.476616	10.46236	8.811488	9.583487	10.55506	10.42372	10.1601	10.37963	0.796141
hsa-miR-34b-5p	5.951029	7.204513	6.090935	6.415492	6.991368	6.825472	6.430519	6.749119	0.333627
hsa-miR-34c-5p	3.881039	4.8823	4.655439	4.472926	3.897095	4.035594	3.615854	3.849514	-0.62341
hsa-miR-361-3p	3.747817	4.225069	3.885433	3.952773	3.613617	3.512674	3.852817	3.659703	-0.29307
hsa-miR-361-5p	6.783265	7.001804	6.688065	6.824378	6.773559	6.708653	7.392281	6.958164	0.133786
hsa-miR-362-3p	3.051007	3.756384	3.455643	3.421011	3.241227	3.376172	2.736417	3.117939	-0.30307
hsa-miR-362-5p	3.615192	4.14128	3.836135	3.864203	2.936446	3.597308	3.291531	3.275095	-0.58911
hsa-miR-3648	3.059734	3.293265	3.00064	3.11788	2.94339	4.928963	2.906491	3.592948	0.475068
hsa-miR-3651	6.463164	7.359977	6.624091	6.815744	7.058732	7.531135	8.400697	7.663522	0.847777
hsa-miR-3652	3.158251	3.306433	3.144925	3.203203	2.815802	4.390524	4.35254	3.852955	0.649752

hsa-miR-3653	3.420548	3.906284	3.723342	3.683391	3.338476	4.269173	3.456821	3.688157	0.004765
hsa-miR-3656	4.505705	4.570451	4.099968	4.392042	5.601597	6.823023	5.349392	5.924671	1.532629
hsa-miR-3659	4.154624	3.439873	3.95841	3.850969	2.913473	3.629712	3.059538	3.200908	-0.65006
hsa-miR-365a-3p	10.6102	10.59285	10.56754	10.5902	10.79915	10.64314	10.61599	10.68609	0.095893
hsa-miR-3663-3p	3.413831	5.65139	3.657602	4.240941	6.853649	7.804517	3.9503	6.202822	1.961881
hsa-miR-3665	7.060302	7.845249	6.886986	7.264179	9.709871	10.72875	8.286845	9.575156	2.310977
hsa-miR-3679-5p	5.054096	4.378877	4.673086	4.70202	3.828175	5.530515	5.458356	4.939015	0.236996
hsa-miR-3682-3p	2.764998	2.705767	2.758732	2.743166	2.619224	3.813119	2.831196	3.087846	0.34468
hsa-miR-369-5p	4.242275	4.032113	3.820774	4.031721	4.713338	3.756612	3.451908	3.973953	-0.05777
hsa-miR-370	4.071959	3.459008	3.678176	3.736381	2.978967	3.511924	3.694581	3.395157	-0.34122
hsa-miR-371a-5p	3.520252	4.16079	3.68198	3.787674	4.864344	6.484361	4.469704	5.272803	1.485129
hsa-miR-373-5p	2.615926	2.91066	2.785732	2.770773	2.761563	3.400051	2.737573	2.966396	0.195623
hsa-miR-374a-5p	6.766827	7.301372	7.217544	7.095248	6.764339	6.334638	5.328076	6.142351	-0.9529
hsa-miR-374b-5p	5.512695	6.06188	5.851177	5.808584	5.589472	5.483243	5.30023	5.457648	-0.35094
hsa-miR-376a-3p	7.997216	7.483141	7.562804	7.681054	8.10768	7.028292	6.725815	7.287262	-0.39379
hsa-miR-376a-5p	4.796607	4.488408	4.219407	4.501474	4.504264	3.967184	3.528961	4.000136	-0.50134
hsa-miR-376b	5.200194	4.481132	5.127599	4.936308	3.827558	3.613705	3.313414	3.584892	-1.35142
hsa-miR-376c	8.358201	7.860919	7.909343	8.042821	8.387613	7.37186	7.150244	7.636572	-0.40625
hsa-miR-377-3p	7.58253	7.211892	7.364509	7.38631	7.655644	6.455024	6.305716	6.805461	-0.58085
hsa-miR-377-5p	3.408466	3.058265	3.178953	3.215228	3.60246	3.034143	3.146461	3.261022	0.045794
hsa-miR-379-5p	6.924517	5.911931	6.316977	6.384475	6.136491	5.424497	6.418764	5.993251	-0.39122
hsa-miR-381	5.877	4.999469	5.921076	5.599182	4.82273	3.76383	3.959708	4.182089	-1.41709
hsa-miR-382-5p	5.795678	4.936676	4.97405	5.235468	5.44044	4.668833	5.800661	5.303311	0.067843
hsa-miR-3911	3.540949	3.317252	3.445728	3.434643	2.964087	3.496458	3.069734	3.17676	-0.25788
hsa-miR-3937	2.729704	3.052516	2.910729	2.89765	3.000009	4.134469	2.997713	3.377397	0.479747
hsa-miR-409-3p	7.386977	6.235346	6.738231	6.786851	7.257876	6.554775	7.297188	7.036613	0.249762
hsa-miR-409-5p	5.242936	4.641123	4.452899	4.778986	5.60302	4.596753	4.659229	4.953	0.174014
hsa-miR-410	6.017997	5.415231	5.412352	5.615193	5.975735	5.179486	4.962499	5.372574	-0.24262
hsa-miR-411-5p	4.459519	4.166671	4.065305	4.230498	3.514181	3.508738	3.252586	3.425168	-0.80533
hsa-miR-423-5p	4.300875	4.612431	4.572249	4.495185	3.858884	4.385567	4.688111	4.310854	-0.18433
hsa-miR-424-5p	8.154134	8.908283	8.523061	8.528493	8.985199	7.753939	6.951453	7.896864	-0.63163
hsa-miR-425-5p	5.043526	5.36004	5.445185	5.282917	4.606234	4.724456	4.785041	4.705244	-0.57767
hsa-miR-4257	3.627589	3.556772	3.15242	3.445593	3.082795	5.174426	3.666131	3.974451	0.528857
hsa-miR-4270	3.461495	3.789123	3.252187	3.500935	3.434409	5.648253	3.656636	4.246433	0.745498
hsa-miR-4271	4.117882	5.173711	4.118351	4.469982	5.687142	6.596751	5.44451	5.909468	1.439486
hsa-miR-4281	7.7232	8.936223	7.334926	7.998116	10.01899	10.66142	8.34332	9.674575	1.676459
hsa-miR-4284	11.06971	10.80078	11.18557	11.01869	10.49407	10.91606	11.73307	11.04773	0.029044

hsa-miR-4286	9.936552	9.408142	9.945093	9.763262	9.272707	9.125596	9.405911	9.268071	-0.49519
hsa-miR-4291	4.801385	5.480492	4.521761	4.934546	5.020913	5.41663	4.349511	4.929018	-0.00553
hsa-miR-4298	3.383759	3.152096	3.385251	3.307035	2.625782	3.874452	3.65495	3.385061	0.078026
hsa-miR-4299	7.51466	6.745096	6.962713	7.074157	6.880547	7.011823	7.641562	7.177978	0.103821
hsa-miR-4306	6.087792	6.532836	6.331881	6.317503	5.964448	6.422251	5.678305	6.021668	-0.29584
hsa-miR-4313	3.827533	3.716849	3.576958	3.707113	2.95716	3.284491	3.638462	3.293371	-0.41374
hsa-miR-431-5p	4.531574	3.758237	3.910505	4.066772	3.829982	3.434407	3.241923	3.502104	-0.56467
hsa-miR-4317	3.987566	4.462016	3.830899	4.093494	4.113336	4.186021	3.958581	4.085979	-0.00751
hsa-miR-4324	3.768618	4.367955	3.788158	3.97491	4.479657	4.05441	4.034719	4.189595	0.214685
hsa-miR-432-5p	5.685795	4.947911	4.691376	5.108361	5.918478	5.520806	5.724246	5.721177	0.612816
hsa-miR-4327	3.649166	4.997746	3.700531	4.115814	6.06136	6.820775	4.870091	5.917409	1.801594
hsa-miR-450a-5p	4.536669	5.385428	5.004533	4.975543	4.714252	4.124319	3.600057	4.146209	-0.82933
hsa-miR-454-3p	3.616471	3.927389	3.661947	3.735269	2.924463	3.486847	3.499813	3.303707	-0.43156
hsa-miR-455-3p	6.337275	6.546136	7.295451	6.726287	6.122289	5.048113	7.376569	6.182324	-0.54396
hsa-miR-483-5p	3.072982	3.113611	3.052293	3.079629	2.50931	3.496458	3.114059	3.039942	-0.03969
hsa-miR-484	4.482888	4.541328	4.609549	4.544588	3.9903	3.803747	4.26788	4.020642	-0.52395
hsa-miR-485-3p	4.575412	3.635028	3.997821	4.06942	4.935673	3.731296	4.393364	4.353444	0.284024
hsa-miR-487b	6.490673	5.667974	5.98474	6.047796	6.592738	5.401071	5.689162	5.894324	-0.15347
hsa-miR-493-5p	7.128359	6.546749	6.313503	6.662871	7.047317	6.399316	6.791869	6.746167	0.083297
hsa-miR-494	8.57282	7.675912	8.392799	8.213844	7.772796	7.947194	8.468601	8.062864	-0.15098
hsa-miR-495	6.31608	5.588104	5.663184	5.855789	6.281227	5.589193	5.390579	5.753666	-0.10212
hsa-miR-497-5p	4.831235	6.075163	5.124638	5.343678	4.978905	4.89493	4.483737	4.785857	-0.55782
hsa-miR-498	3.478613	3.398013	3.235416	3.370681	3.599275	3.857843	3.691806	3.716308	0.345627
hsa-miR-500a-3p	3.390477	3.600696	3.379389	3.456854	3.119725	3.408144	3.202097	3.243322	-0.21353
hsa-miR-501-5p	3.176231	3.302425	3.3374	3.272019	3.008404	3.102005	3.45671	3.18904	-0.08298
hsa-miR-502-3p	3.398454	3.676037	3.183755	3.419415	2.969568	3.321444	2.91518	3.06873	-0.35068
hsa-miR-503	3.77934	3.994412	4.303001	4.025584	3.083869	3.267219	3.100655	3.150581	-0.875
hsa-miR-505-3p	4.873423	4.963457	4.923317	4.920066	4.339725	4.49948	5.084107	4.641104	-0.27896
hsa-miR-505-5p	3.348641	3.390196	3.180607	3.306481	2.833243	3.4824	3.434372	3.250005	-0.05648
hsa-miR-513a-5p	3.744735	3.398013	3.626621	3.58979	2.849232	3.848601	3.793169	3.497001	-0.09279
hsa-miR-513b	3.136658	2.856323	2.783655	2.925545	2.62244	3.01293	2.866323	2.833898	-0.09165
hsa-miR-532-3p	3.456159	3.718704	3.330096	3.501653	3.744429	3.704489	3.625331	3.691417	0.189764
hsa-miR-532-5p	3.9838	4.364967	3.866902	4.07189	3.400404	4.124082	3.734013	3.752833	-0.31906
hsa-miR-539-5p	4.490304	3.663638	3.696682	3.950208	3.72353	3.641173	3.95841	3.774371	-0.17584
hsa-miR-542-3p	4.343962	5.300993	4.743165	4.79604	3.920628	4.20418	3.251538	3.792115	-1.00392
hsa-miR-542-5p	4.103694	4.711171	4.176074	4.330313	3.939699	4.217653	4.051788	4.069713	-0.2606
hsa-miR-543	5.650354	4.30012	4.813232	4.921236	4.803146	4.395126	5.331822	4.843365	-0.07787

hsa-miR-551b-3p	3.44877	4.863133	3.905287	4.072397	4.833095	3.694495	4.289997	4.272529	0.200132
hsa-miR-572	3.409158	3.188511	3.197372	3.265013	3.24014	4.709856	3.714189	3.888062	0.623048
hsa-miR-574-3p	5.781708	6.359074	5.784381	5.975055	6.586657	6.188753	6.649395	6.474935	0.49988
hsa-miR-574-5p	6.346157	6.135412	6.465336	6.315635	5.890416	5.847179	6.557478	6.098358	-0.21728
hsa-miR-575	5.437104	4.736699	5.058076	5.077293	4.384177	5.317518	5.822559	5.174751	0.097458
hsa-miR-590-5p	4.417244	4.716257	5.507386	4.880295	3.060935	3.55601	3.120042	3.245662	-1.63463
hsa-miR-615-3p	2.999142	3.163869	2.949696	3.037569	3.549266	2.716758	2.896832	3.054285	0.016716
hsa-miR-625-5p	3.462987	3.429895	3.584605	3.492496	2.712785	2.981723	3.618397	3.104302	-0.38819
hsa-miR-629-3p	2.760827	2.999118	2.924416	2.894787	3.076887	3.83988	2.833452	3.250073	0.355286
hsa-miR-629-5p	2.663405	2.830092	2.831855	2.775117	2.485886	2.663691	2.876154	2.675244	-0.09987
hsa-miR-630	7.121106	6.161774	6.757265	6.680048	6.692072	6.948518	6.608298	6.749629	0.069581
hsa-miR-636	2.795271	2.768872	2.968755	2.844299	3.159175	3.782715	3.038112	3.326667	0.482368
hsa-miR-638	6.372137	5.79093	5.956719	6.039929	6.795265	8.557768	6.84302	7.398684	1.358756
hsa-miR-642b-3p	3.857028	4.29292	3.873284	4.007744	4.490651	6.619138	4.392816	5.167535	1.159791
hsa-miR-654-3p	6.56643	6.016096	6.129415	6.237313	6.664626	5.427403	5.990164	6.027398	-0.20992
hsa-miR-660-5p	3.970664	4.475859	4.087451	4.177992	3.949013	3.820235	3.165862	3.645037	-0.53295
hsa-miR-663a	2.895696	2.969218	2.77956	2.881491	2.886934	4.337866	2.895811	3.373537	0.492046
hsa-miR-664-3p	3.486989	4.003536	3.409744	3.633423	5.134	4.527856	4.113665	4.59184	0.958418
hsa-miR-671-5p	3.205063	3.12226	3.025933	3.117752	2.79011	3.88512	3.158723	3.277984	0.160232
hsa-miR-718	2.64762	3.042432	2.637483	2.775845	2.818966	3.734397	2.704163	3.085842	0.309997
hsa-miR-720	14.31532	13.55716	14.31744	14.06331	13.39261	13.07101	13.69184	13.38515	-0.67816
hsa-miR-744-5p	3.812056	3.678822	3.762387	3.751088	3.059976	3.886531	3.531947	3.492818	-0.25827
hsa-miR-758	5.59246	4.304339	5.198297	5.031699	4.95874	3.970343	4.674789	4.534624	-0.49707
hsa-miR-7-5p	5.3219	4.186969	6.176139	5.228336	3.008593	3.306889	4.107645	3.474375	-1.75396
hsa-miR-762	5.106899	5.654745	5.186092	5.315912	8.090597	10.27974	6.060762	8.143701	2.827788
hsa-miR-769-5p	3.508177	3.789675	3.372279	3.55671	3.143905	3.502293	3.374217	3.340138	-0.21657
hsa-miR-874	3.967716	4.412333	3.783154	4.054401	5.105962	6.999606	4.805375	5.636981	1.58258
hsa-miR-887	2.744663	2.735292	2.556069	2.678675	3.08424	3.089777	2.587222	2.920413	0.241738
hsa-miR-892b	3.839438	3.743715	3.764253	3.782469	3.255389	3.63464	4.484727	3.791585	0.009117
hsa-miR-92a-3p	7.114861	6.926419	7.406127	7.149136	5.958289	5.210886	7.571024	6.246733	-0.9024
hsa-miR-93-5p	6.327971	6.227078	7.054318	6.536455	4.640164	5.159068	6.35662	5.385284	-1.15117
hsa-miR-939	4.728064	4.486468	4.775015	4.663182	5.283754	6.17211	5.059711	5.505191	0.842009
hsa-miR-940	5.543792	5.529125	5.224862	5.432593	5.554167	7.192472	5.982197	6.242945	0.810353
hsa-miR-98	6.205319	6.615555	6.432265	6.417713	6.211883	6.430272	6.368407	6.336854	-0.08086
hsa-miR-99a-5p	6.951022	7.418073	7.513476	7.29419	8.048218	7.397531	7.384609	7.610119	0.315929
hsa-miR-99b-5p	7.254139	7.297411	7.184146	7.245232	7.251552	7.365712	7.32183	7.313031	0.067799

SUPPLEMENTAL TABLE S2

miRNA	number of predicted targets	Cell-mediated Immune Response	Cell Cycle	Cellular Assembly and Organization	Cellular Growth and Proliferation	Lipid Metabolism	Tissue Development
Group A c8-c4							
miR-1207	2359	4.47E-03-1.99E-02<br>14	1.5E-04-2.14E-02<br>114	3.63E-06-2.14E-02<br>272	4.12E-06-2.14E-02<br>558	1.48E-03-2.14E-02<br>30	2.16E-04-2.1E-02<br>221
miR-1225-5p	504	3.97E-03-2.42E-02<br>8	1.46E-03-2.42E-02<br>13	8.22E-03-2.42E-02<br>52	5.84E-04-2.42E-02<br>41	4.89E-06-2.42E-02<br>11	2.63E-03-2.42E-02<br>34
miR-17-5p	1419	4.77E-03-4.77E-03<br>4	1.05E-07-6.68E-03<br>175	1.03E-06-7.17E-03<br>181	1.97E-08-7.08E-03<br>369	1.25E-03-4.77E-03<br>8	4.81E-06-7.08E-03<br>222
miR-1915-3p	1813	5.55E-03-1.7E-02<br>16	8.41E-03-2.09E-02<br>10	5.45E-04-2.09E-02<br>169	2E-03-2.09E-02<br>78	2.09E-02-2.09E-02<br>4	5.36E-04-2.09E-02<br>114
miR-2861	1664	7.11E-03-1.73E-02<br>8	6.09E-03-4.68E-02<br>51	2.7E-06-4.63E-02<br>186	7.11E-03-4.44E-02<br>86	4.75E-04-3.28E-02<br>36	1.79E-03-4.68E-02<br>126
miR-293-5p	545	NA<br>NA	9.56E-03-3.59E-02<br>41	1.55E-04-3.03E-02<br>75	8.96E-03-2.62E-02<br>132	2.98E-05-3.21E-02<br>13	7.74E-03-3.21E-02<br>30
miR-3665	892	1.19E-03-4.21E-02<br>9	2.89E-03-4.22E-02<br>18	9.65E-09-4.21E-02<br>124	7.04E-04-4.21E-02<br>226	1.78E-03-4.21E-02<br>12	1.26E-03-4.22E-02<br>68
miR-4281	677	5.96E-03-3.22E-02<br>6	3E-04-3.22E-02<br>21	4.54E-04-3.22E-02<br>72	1.43E-03-3.22E-02<br>13	3.05E-03-3.22E-02<br>11	1.04E-03-3.22E-02<br>13
miR-4327	542	6.44E-03-2.61E-02<br>5	5.17E-04-2.61E-02<br>65	2.77E-04-2.61E-02<br>62	2.56E-04-2.61E-02<br>27	6.78E-04-2.61E-02<br>16	4.31E-04-2.61E-02<br>56
miR-762	2172	4.81E-03-2.79E-02<br>13	4.25E-03-3.07E-02<br>61	7.65E-06-3.07E-02<br>265	2.32E-05-3.07E-02<br>508	1.52E-03-3.07E-02<br>62	3.47E-05-3.07E-02<br>161
miR-92a-3p	1140	2.22E-03-7.6E-03<br>8	1.85E-04-2.72E-02<br>125	6.53E-08-2.72E-02<br>202	2.08E-05-2.72E-02<br>284	6.24E-03-2.72E-02<br>14	6.87E-07-2.67E-02<br>179
Group B o8-o4							
miR-138-5p	1201	2.95E-02-3.54E-02<br>5	1.91E-04-3.54E-02<br>126	8.98E-04-3.47E-02<br>141	1.79E-04-3.58E-02<br>290	5.61E-03-3.54E-02<br>17	5.17E-04-3.58E-02<br>123
miR-16-5p	2020	NA<br>NA	1.2E-12-5.8E-03<br>244	1.75E-08-5.65E-03<br>271	1.94E-06-6.13E-03<br>493	3.44E-03-4.59E-03<br>10	2.17E-05-5.8E-03<br>244
miR-181a-5p	1498	4.06E-03-4.06E-03<br>7	4.61E-06-8.27E-03<br>161	2.52E-06-7.4E-03<br>173	1.89E-08-8.62E-03<br>399	1.55E-03-6.55E-03<br>12	1.26E-05-8.62E-03<br>194
miR-18a-5p	696	2.45E-03-1.11E-02<br>10	8.85E-05-1.36E-02<br>75	4.12E-05-1.33E-02<br>70	1.03E-04-1.46E-02<br>164	1.03E-02-1.03E-02<br>2	4.71E-04-1.44E-02<br>64
miR-19a-3p	1450	5E-03-5.49E-03<br>4	5.14E-04-1.06E-02<br>119	7.8E-07-1.43E-02<br>195	2.77E-07-1.15E-02<br>382	1.34E-03-1.43E-02<br>14	2.98E-05-1.38E-02<br>232
miR-21-3p	712	3.42E-02-3.42E-02<br>2	6.97E-04-3.42E-02<br>81	3.67E-04-3.42E-02<br>71	2.27E-04-3.42E-02<br>178	3.95E-03-3.42E-02<br>30	7.53E-04-3.42E-02<br>79

miR-21-5p	617	1.42E-03-3.54E-03<br>23	2.67E-05-3.76E-03<br>67	6.98E-05-3.7E-03<br>77	3.66E-06-3.53E-03<br>175	2.01E-04-2.8E-03<br>19	4.24E-06-1.96E-03<br>91
miR-218-5p	1279	NA<br>NA	5.55E-04-2.11E-02<br>110	3.68E-06-2.57E-02<br>156	2.47E-04-2.11E-02<br>300	1.1E-02-2.11E-02<br>7	5.15E-07-2.11E-02<br>143
miR-23a-3p	1506	NA<br>NA	3.8E-04-1.61E-02<br>139	2.52E-05-1.97E-02<br>167	3.7E-06-1.97E-02<br>374	5.25E-03-1.5E-02<br>7	7.15E-10-1.9E-02<br>157
miR-30a-3p	14	7.07E-04-2.59E-02<br>3	1.41E-03-3.96E-02<br>4	7.07E-04-4.16E-02<br>6	9.36E-04-4.3E-02<br>8	1.41E-03-4.94E-03<br>2	7.07E-04-4.3E-02<br>6
miR-31-5p	952	3E-03-3.41E-02<br>7	2.73E-04-3.01E-02<br>49	2.11E-04-3.27E-02<br>100	1.15E-03-1.99E-02<br>85	3.8E-04-1.2E-02<br>8	8.14E-04-2.64E-02<br>90
miR-3118	731	NA<br>NA	1.18E-03-3.44E-02<br>28	1.87E-05-3.44E-02<br>58	1.99E-03-3.44E-02<br>64	1.52E-02-3.44E-02<br>20	4.05E-05-3.44E-02<br>40
miR-3656	470	9.36E-03-2.19E-02<br>3	4.59E-03-2.19E-02<br>38	4.8E-04-2.19E-02<br>57	6.8E-04-2.19E-02<br>40	4.31E-03-2.19E-02<br>13	4.59E-03-2.19E-02<br>48
miR-4271	1574	1.21E-02-4.92E-02<br>7	1.64E-03-4.92E-02<br>70	1.36E-04-4.92E-02<br>196	2.28E-03-4.92E-02<br>85	3.87E-03-1.63E-02<br>3	9.54E-04-4.92E-02<br>121
miR-503-5p	772	NA<br>NA	2.49E-11-7.8E-03<br>106	1.12E-05-7.8E-03<br>99	6.58E-06-7.8E-03<br>199	1.58E-03-7.8E-03<br>12	2.5E-06-7.8E-03<br>105
miR-664-3p	242	1.15E-02-1.15E-02<br>3	3.93E-04-2.29E-02<br>8	6.38E-03-2.29E-02<br>25	8.12E-05-2.29E-02<br>21	1.92E-03-2.29E-02<br>9	4.42E-04-2.29E-02<br>20
miR-708-5p	875	1.84E-02-4.14E-02<br>5	1.71E-03-4.14E-02<br>23	3.65E-03-4.14E-02<br>88	1.71E-03-4.14E-02<br>42	1.54E-03-4.06E-02<br>18	2.4E-04-4.14E-02<br>91
miR-7a-5p	917	1.08E-02-1.75E-02<br>2	6.75E-04-1.75E-02<br>79	4.36E-05-1.75E-02<br>142	2.54E-05-1.75E-02<br>216	6.84E-03-1.08E-02<br>10	5.94E-05-1.75E-02<br>92
Group C y8-y4							
miR-1181	94	NA<br>NA	1.47E-03-4.26E-02<br>11	4.34E-03-3.84E-02<br>8	2.3E-04-4.84E-02<br>28	4.34E-03-3E-02<br>4	4.34E-03-4.84E-02<br>12
miR-125-5b	1532	1.16E-03-1.55E-02<br>30	3.88E-04-2.02E-02<br>88	3.97E-04-1.96E-02<br>33	1.06E-04-1.68E-02<br>354	1.35E-03-5.41E-03<br>10	3.17E-04-1.74E-02<br>112
miR-130a-3p	1253	3.73E-03-3.73E-03<br>2	4.89E-06-3.95E-03<br>134	5.44E-07-3.73E-03<br>179	9.96E-08-3.68E-03<br>335	2.85E-04-3.95E-03<br>26	8.23E-07-3.91E-03<br>216
miR-151-3p	349	1.67E-02-3.31E-02<br>2	1.67E-02-4.92E-02<br>8	3.98E-03-4.92E-02<br>44	1.46E-02-4.92E-02<br>32	3.98E-03-4.92E-02<br>9	3.51E-04-4.92E-02<br>42
miR-155-5p	799	9.2E-04-1.13E-02<br>22	1.97E-07-1.12E-02<br>94	1.37E-05-1.12E-02<br>103	6.35E-05-1.26E-02<br>207	4.45E-03-8.67E-03<br>4	1.05E-04-1.13E-02<br>102
miR-1908-5p	1212	1.48E-04-1.48E-04<br>4	7.26E-04-2.94E-02<br>95	2.93E-05-2.97E-02<br>152	3.31E-04-3.04E-02<br>290	5.57E-03-2.97E-02<br>20	2.98E-05-2.99E-02<br>159
miR-193a-5p	554	2.61E-02-2.61E-02<br>2	2E-03-2.61E-02<br>16	2E-03-2.66E-02<br>24	6.46E-03-2.66E-02<br>25	6.78E-04-2.66E-02<br>28	2E-03-2.66E-02<br>17
miR-196a-5p	629	1.98E-03-2.82E-02<br>5	3.29E-04-3.01E-02<br>33	2.08E-03-2.82E-02<br>70	2.48E-03-2.82E-02<br>53	1.25E-02-2.69E-02<br>9	2.08E-03-2.81E-02<br>54

miR-21-5p	617	1.42E-03-3.54E-03<br>23	2.67E-05-3.76E-03<br>67	6.98E-05-3.7E-03<br>77	3.66E-06-3.53E-03<br>175	2.01E-04-2.8E-03<br>19	4.24E-06-1.96E-03<br>91
miR-3162-5p	573	1.51E-02-2.81E-02<br>4	4.57E-03-2.81E-02<br>15	2.33E-03-2.81E-02<br>52	3.66E-03-2.81E-02<br>22	2.01E-02-2.81E-02<br>10	2.1E-03-2.81E-02<br>39
miR-3180-3p	1102	8E-03-2.48E-02<br>5	1.24E-03-2.48E-02<br>45	2.55E-07-2.48E-02<br>154	7.74E-04-2.48E-02<br>261	1.01E-03-1.54E-02<br>21	3.04E-08-2.48E-02<br>124
miR-320b	1074	6.34E-03-1.71E-02<br>6	4.56E-05-2.45E-02<br>115	1.16E-03-2.45E-02<br>45	4.51E-05-2.45E-02<br>263	1.29E-02-1.84E-02<br>9	4.36E-04-2.45E-02<br>84
miR-3547	742	1.52E-02-3.59E-02<br>6	9.27E-05-3.59E-02<br>41	1.85E-05-3.59E-02<br>53	1.29E-03-3.59E-02<br>169	7.37E-03-3.59E-02<br>17	4.45E-04-3.59E-02<br>33
miR-3648	286	4.85E-03-4.41E-02<br>13	5.57E-03-4.46E-02<br>31	3.56E-03-4.75E-02<br>27	2.67E-02-2.67E-02<br>1	2.67E-02-4.73E-02<br>3	NA<br>NA
miR-409-3p	189	8.88E-03-4.36E-02<br>3	8.99E-03-3.55E-02<br>9	1.18E-03-4.58E-02<br>15	4.19E-03-4.42E-02<br>7	4.19E-03-4.8E-02<br>7	2.4E-04-4.42E-02<br>15
miR-574-5p	565	NA<br>NA	2.1E-03-2.67E-02<br>8	1.49E-04-2.67E-02<br>62	7.13E-04-2.67E-02<br>13	1.79E-02-2.67E-02<br>9	1.49E-04-2.67E-02<br>30
miR-642-3p	512	1.79E-02-2.45E-02<br>4	1.63E-03-2.45E-02<br>45	7.02E-06-2.45E-02<br>60	3.33E-04-2.45E-02<br>33	5.97E-04-2.45E-02<br>17	2.07E-03-2.45E-02<br>31
miR-660-5p	481	3E-02-3E-02<br>2	3.66E-03-4.55E-02<br>20	2.05E-03-3.55E-02<br>34	2.14E-02-4.55E-02<br>17	1.56E-03-4.55E-02<br>23	2.3E-02-4.45E-02<br>21
miR-718	242	1.14E-02-1.14E-02<br>1	1.08E-02-4.49E-02<br>12	1.89E-03-4.57E-02<br>38	5.21E-03-4.49E-02<br>25	1.14E-02-4.86E-02<br>15	3.51E-03-4.76E-02<br>27
miR-874	1070	NA<br>NA	1.17E-04-2.12E-02<br>86	3.11E-05-2.39E-02<br>126	1.6E-06-2.45E-02<br>252	7.7E-03-1.65E-02<br>5	1.6E-06-2.45E-02<br>122
miR-879-5p	683	9.99E-03-2.62E-02<br>4	1.25E-04-3.27E-02<br>35	6.35E-04-2.9E-02<br>63	2.98E-04-3.27E-02<br>34	6.13E-03-3.27E-02<br>16	5.46E-04-3.27E-02<br>39

SUPPLEMENTAL TABLE S3

Gene	Group A c8-c4	Group B o8-o4	Group C y8-y4
BHLHE41	hsa-miR-93-5p/miR-17-5p	hsa-miR-15b-5p/miR-16-5p<br>hsa-miR-20a-5p/miR-17-5p	hsa-miR-130b-3p/miR-130a-3p<br>hsa-miR-320a/mir-320b
CCND1	hsa-miR-93-5p/miR-17-5p	hsa-miR-15b-5p/miR-16-5p<br>hsa-miR-20a-5p/miR-17-5p<br>hsa-miR-19a-3p<br>hsa- miR-503	hsa-miR-155-5p
CMTM4	hsa-miR-93-5p/miR-17-5p	hsa-miR-15b-5p/miR-16-5p<br>hsa-miR-20a-5p/miR-17-5p<br>hsa-miR-503	hsa-miR-125a-5p<br>hsa- miR-320a
CYLD	hsa-miR-93-5p/miR-17-5p	hsa-miR-15b-5p/miR-16-5p<br>hsa-miR-20a-5p/miR-17-5p<br>hsa-miR-19a-3p/miR-19b-3p	hsa-miR-130b-3p<br>hsa- miR-320a
FAM46B	hsa-miR-1207<br>hsa-miR-1915<br>hsa- miR-2861<br>hsa-miR-3665<br>hsa- miR-762		
HMGB2	hsa-miR-371a-5p/miR-293-5p	hsa-miR-181a-5p<br>hsa-miR-23b-3p/miR-23a-3p	
HNRNPH1	hsa-miR-2861		
LBR	hsa-miR-4327	hsa-miR-181a-5p<br>hsa-miR-23b-3p	
OLFML3	hsa-miR-2861		
RMI1	hsa-miR-1225-5p		
SOCS3	hsa-miR-2861<br>hsa-miR-762	hsa-miR-181-5p	hsa-miR-718<br>hsa-miR-874/miR-874-3p

SUPPLEMENTAL TABLE S4: Expression of members of miR17/92 cluster and its target mRNAs in BM-MSCs. Experimentally validated target mRNAs of the miR-17-92 cluster an its two paralogues miR106a/363 and miR-106b/25 are shown.

MiRNA	Genomic location	Group	miRNA expression	target	target expression	ref
**hsa-miR-17-5p**	miR17/92	o8-o4	-1.77	RUNX1	1.09	[1, 2]
**hsa-miR-17-5p**	miR17/92	o8-o4	-1.77	NCOA3		[1, 3]
**hsa-miR-17-5p**	miR17/92	o8-o4	-1.77	PCAF		[1]
**hsa-miR-17-5p**	miR17/92	o8-o4	-1.77	TGFBR2		[1]
**hsa-miR-17-5p**	miR17/92	o8-o4	-1.77	BCL2L11	-	[1]
**hsa-miR-17-5p**	miR17/92	o8-o4	-1.77	CCND1	0.70	[1, 2]
**hsa-miR-17-5p**	miR17/92	o8-o4	-1.77	CDKN1A (p21)		[1, 4]
**hsa-miR-17-5p**	miR17/92	o8-o4	-1.77	GAP1		[1]
**hsa-miR-17-5p**	miR17/92	o8-o4	-1.77	IRF1		[1]
**hsa-miR-17-5p**	miR17/92	o8-o4	-1.77	MAPK9		[1]
**hsa-miR-17-5p**	miR17/92	o8-o4	-1.77	MAPK14		[5]
**hsa-miR-17-5p**	miR17/92	o8-o4	-1.77	PKNOX1		[6]
**hsa-miR-17-5p**	miR17/92	o8-o4	-1.77	Eos		[7]
**hsa-miR-17-5p**	miR17/92	o8-o4	-1.77	NR4A3		[1]
**hsa-miR-17-5p**	miR17/92	o8-o4	-1.77	MYCN		[1]
**hsa-miR-17-5p**	miR17/92	o8-o4	-1.77	TSG01		[1]
**hsa-miR-20a-5p**	miR17/92	o8-o4	-1.80	PCAF		[1]
**hsa-miR-20a-5p**	miR17/92	o8-o4	-1.80	RUNX1	1.09	[1, 2]
**hsa-miR-20a-5p**	miR17/92	o8-o4	-1.80	TGFBR2		[1, 8]
**hsa-miR-20a-5p**	miR17/92	o8-o4	-1.80	E2F1	-1.64	[9, 10]
**hsa-miR-20a-5p**	miR17/92	o8-o4	-1.80	CCND1	0.70	[2]
**hsa-miR-20a-5p**	miR17/92	o8-o4	-1.80	MAPK14		[5]
**hsa-miR-106b-5p**	miR106b/25	o8-o4		ITCH		[11]
**hsa-miR-106b-5p**	miR106b/25	o8-o4		MAPK14		[5]
**hsa-miR-106b-5p**	miR106b/25	o8-o4	-1,55	CDKN1A (p21)		[4]
**hsa-miR-93-5p**	miR-106b/25	c8-c4	-1.15 (o8-o4) -0.96 (y8-y4	ITGB8		[12]
**hsa-miR-18a**	miR17/92	o8-o4	-1.67	CTGF		[1, 13]
**hsa-miR-19a-3p**	miR17/92	o8-o4	-2.25	PKNOX1		[6]
**hsa-miR-363**	miR106a/363			MYO1B		[14]

REFERENCES

1. Cloonan N, Brown MK, Steptoe AL, Wani S, Chan WL, Forrest AR, Kolle G, Gabrielli B, Grimmond SM. The miR-17-5p microRNA is a key regulator of the G1/S phase cell cycle transition. Genome Biol. 2008; 9:R127-2008-9-8-r127. Epub 2008 Aug 14.

2. Yu Z, Wang C, Wang M, Li Z, Casimiro MC, Liu M, Wu K, Whittle J, Ju X, Hyslop T, McCue P, Pestell RG. A cyclin D1/microRNA 17/20 regulatory feedback loop in control of breast cancer cell proliferation. J Cell Biol. 2008; 182:509-517.

3. Hossain A, Kuo MT, Saunders GF. Mir-17-5p regulates breast cancer cell proliferation by inhibiting translation of AIB1 mRNA. Mol Cell Biol. 2006; 26:8191-8201.

4. Ivanovska I, Ball AS, Diaz RL, Magnus JF, Kibukawa M, Schelter JM, Kobayashi SV, Lim L, Burchard J, Jackson AL, Linsley PS, Cleary MA. MicroRNAs in the miR-106b family regulate p21/CDKN1A and promote cell cycle progression. Mol Cell Biol. 2008; 28:2167-2174.

5. Carraro G, El-Hashash A, Guidolin D, Tiozzo C, Turcatel G, Young BM, De Langhe SP, Bellusci S, Shi W, Parnigotto PP, Warburton D. miR-17 family of microRNAs controls FGF10-mediated embryonic lung epithelial branching morphogenesis through MAPK14 and STAT3 regulation of E-cadherin distribution. Dev Biol. 2009; 333:238-250.

6. Mian YA, Zeleznik-Le NJ. The miR-17~92 cluster contributes to MLL leukemia through the repression of MEIS1 competitor PKNOX1. Leuk Res. 2016; 46:51-60.

7. Yang HY, Barbi J, Wu CY, Zheng Y, Vignali PD, Wu X, Tao JH, Park BV, Bandara S, Novack L, Ni X, Yang X, Chang KY et al. MicroRNA-17 modulates regulatory T cell function by targeting co-regulators of the Foxp3 transcription factor. Immunity. 2016; 45:83-93.

8. Volinia S, Calin GA, Liu CG, Ambs S, Cimmino A, Petrocca F, Visone R, Iorio M, Roldo C, Ferracin M, Prueitt RL, Yanaihara N, Lanza G et al. A microRNA expression signature of human solid tumors defines cancer gene targets. Proc Natl Acad Sci U S A. 2006; 103:2257-2261.

9. O'Donnell KA, Wentzel EA, Zeller KI, Dang CV, Mendell JT. C-myc-regulated microRNAs modulate E2F1 expression. Nature. 2005; 435:839-843.

10. Pickering MT, Stadler BM, Kowalik TF. miR-17 and miR-20a temper an E2F1-induced G1 checkpoint to regulate cell cycle progression. Oncogene. 2009; 28:140-145.

11. Sampath D, Calin GA, Puduvalli VK, Gopisetty G, Taccioli C, Liu CG, Ewald B, Liu C, Keating MJ, Plunkett W. Specific activation of microRNA106b enables the p73 apoptotic response in chronic lymphocytic leukemia by targeting the ubiquitin ligase itch for degradation. Blood. 2009; 113:3744-3753.

12. Fang L, Deng Z, Shatseva T, Yang J, Peng C, Du WW, Yee AJ, Ang LC, He C, Shan SW, Yang BB. MicroRNA miR-93 promotes tumor growth and angiogenesis by targeting integrin-beta]8. Oncogene. 2011; 30:806-821.

13. Ohgawara T, Kubota S, Kawaki H, Kondo S, Eguchi T, Kurio N, Aoyama E, Sasaki A, Takigawa M. Regulation of chondrocytic phenotype by micro RNA 18a: Involvement of Ccn2/ctgf as a major target gene. FEBS Lett. 2009; 583:1006-1010.

14. Chapman BV, Wald AI, Akhtar P, Munko AC, Xu J, Gibson SP, Grandis JR, Ferris RL, Khan SA. MicroRNA-363 targets myosin 1B to reduce cellular migration in head and neck cancer. BMC Cancer. 2015; 15:861-015-1888-3.
